# Predictive Hydration Model of Portland Cement and Its Main Minerals Based on Dissolution Theory and Water Diffusion Theory

**DOI:** 10.3390/ma14030595

**Published:** 2021-01-27

**Authors:** Tianqi Qi, Wei Zhou, Xinghong Liu, Qiao Wang, Sifan Zhang

**Affiliations:** State Key Laboratory of Water Resources and Hydropower Engineering Science, Wuhan University, Wuhan 430072, China; tianqi_qi@whu.edu.cn (T.Q.); liuxht@126.com (X.L.); qiaowang@whu.edu.cn (Q.W.); sf.zhang@whu.edu.cn (S.Z.)

**Keywords:** portland cement, hydration, water diffusion theory, dissolution theory, C_3_A-gypsum system, prediction

## Abstract

Efficient and accurate cement hydration simulation is an important issue for predicting and analyzing concrete’s performance evolution. A large number of models have been proposed to describe cement hydration. Some models can simulate the test results with high accuracy by constructing reasonable functions, but they are based on mathematical regression and lack of physical background and prediction ability. Other models, such as the famous HYMOSTRUC model and CEMHYD3D model, can predict the hydration rate and microstructure evolution of cement based on its initial microstructure. However, this kind of prediction model also has some limitations, such as the inability to fully consider the properties of cement slurry, or being too complicated for use in finite element analysis (FEA). In this study, the hydration mechanisms of the main minerals in Portland cement (PC) are expounded, and the corresponding hydration model is built. Firstly, a modified particle hydration model of tricalcium silicate (C_3_S) and alite is proposed based on the moisture diffusion theory and the calcium silicate hydrate (C-S-H) barrier layer hypothesis, which can predict the hydration degree of C_3_S and alite throughout the age. Taking the hydration model of C_3_S as a reference, the hydration model of dicalcium silicate (C_2_S) is established, and the synergistic hydration effect of C_3_S and C_2_S is calibrated by analyzing the published test results. The hydration model of tricalcium aluminate(C_3_A)-gypsum system is then designed by combining the theory of dissolution and diffusion. This model can reflect the hydration characteristics of C_3_A in different stages, and quantify the response of the hydration process of C_3_A to different gypsum content, water–cement ratio, and particle size distribution. Finally, several correction coefficients are introduced into the hydration model of the main mineral, to consider the synergistic hydration effect among the minerals to some extent and realize the prediction of the hydration of PC.

## 1. Introduction

### 1.1. Research Background

Modern mass concrete projects have the characteristics of high strength and fast construction speed, which leads to an increase of the dosage of cementitious materials in concrete, accompanied by the high heat of hydration and high shrinkage. The main risk of mass concrete cracking at early age comes from the temperature gradient formed by the heat of cement hydration inside and outside the structure. Excessive temperature gradient will lead to the formation of temperature cracks in the structure under restricted conditions [1,2]. In the later stage, the structure’s service life depends on the concrete performance and service conditions. Besides heat release, cement hydration also determines the strength, self-shrinkage, and self-drying of concrete [3]. In other words, hydration is the basis for the evolution of concrete properties.

The presence of water is an essential condition for cement hydration. On the one hand, water is involved in the reaction; on the other hand, the reaction can last only when the water’s chemical potential overcomes the activation energy barrier. Investigations have shown that if the relative humidity of the pores between cement particles is lower than 0.75–0.8, the hydration will stop [4,5,6]. However, in the interior of mass concrete, the pore humidity may maintain a high value for a decade. Experiments have confirmed that the autogenous shrinkage of concrete under saturated conditions still exhibits logarithmic growth within 10 years [7], which shows that cement hydration is a long-term phenomenon. Therefore, the improvement of structural safety, the quantification of structural durability attenuation, and the modification of cement-based materials need to be carried out based on the full-age hydration research.

Numerical simulation is an effective method for performance prediction and material design. Since the 20th century, a significant progress toward developing cement hydration models has been made. Ulm and Coussy [8] proposed a thermo-chemo-mechanical model for early-age concrete, in which the normalized affinity is determined as an intrinsic kinetic function. Cervera et al. [9] deduced the analytical expression of normalized affinity based on the free energy theory of thermochemical systems, and established a macroscopic hydration model for engineering purposes. Krstulovic and Dabic [10] developed a conceptual model of cement hydration that can describe nucleation and growth, phase boundary reaction and diffusion, and developed a computer program to determine specific kinetic parameters describing individual hydration processes. Di Luzio and Cusatis [11] dealt with the formulation and validation of a hygro-thermo-chemical model for concrete, simulating early age phenomena, such as self-heating and self-desiccation, with great accuracy suitable. Recently, Zhou et al. [12,13] put forward a multi-scale hydration model suitable for analyzing moisture transport and heat transfer at macroscale and mesoscale. The hydration models mentioned above are widely used and have high accuracy in simulating known results. However, these models are all mathematical regression models, which need to be calibrated before use, and have no ability to predict. Moreover, some models cannot consider the influence of the chemical potential of water on hydration. To master the evolution law of concrete performance in the whole cycle of design, construction, and operation—or to reduce the physical test cost—it is valuable to establish a predictive hydration model. Taking tricalcium silicate (C_3_S) particles as the research object, Jennings and Johnson [14] built a model to simulate the microstructure development during the hydration of C_3_S, which had the potential to predict microstructure and bulk properties. Van breugel [15] and Bentz [16] proposed the famous HYMOSTRUC model and CEMHYD3D model respectively, which appear to be the most accepted ones. Both models can accurately predict the influence of particle size distribution, water state and reaction temperature on hydration rate based on the real initial microstructure of cement, and can describe the microstructure evolution during hydration. However, the complexity of these two models makes it difficult to be applied to the long-term FEA of the structure. Lin and Meyer [17] improved Ulm’s work and proposed a simplified mathematical model to describe and quantify the hydration kinetics. Lin’s model has good prediction ability, but it does not consider the chemical and physical characteristics of cement hydration and the effect of pore moisture reduction on hydration. Rahimi-Aghdam et al. [18] established a long-term hydration model controlled by diffusion through barrier shells of calcium silicate hydrate (C-S-H), which can be applied to the FEA of the structure This model has strong applicability, but there are some limitations: (1) the response of hydration to cement mineral composition cannot be considered; (2) the initial period and dormant period of cement hydration are not described; (3) the model has errors in predicting the long-term hydration of coarse particles.

This work draws on the modeling ideas of Rahimi-Aghdam et al. [18], and proposes a modified hydration model of alite and C_3_S, which can describe the initial period and the dormant period of hydration. By re-calibrating model parameters, the modified model can accurately predict the long-term hydration of coarse particles. Dicalcium silicate (C_2_S) and C_3_S have great similarities in hydration products and reaction mechanisms. Based on the C_3_S hydration model, a predictive hydration model of C_2_S is proposed, which can reflect the hydration characteristics of C_2_S, that is, the early-age hydration rate is low, and the later hydration rate is increased. We also discuss the hydration characteristics and mechanism of tricalcium aluminate (C_3_A)-gypsum system, and divide the reaction of C_3_A-gypsum system into three stages to simulate: (1) C_3_A reacts with gypsum, (2) C_3_A reacts with ettringite, (3) C_3_A reacts directly with water. The hydration model of the first stage is established by applying the dissolution theory, and the hydration models of the remaining two stages are controlled by water passing through the monosulfide hydrated calcium sulphoaluminate (AFm) and calcium aluminates hydrate (C_3_AH_6_) barrier shell. This three-stage hydration model can reflect the influence of gypsum content, water cement ratio (w/c) and particle size distribution on the hydration rate of C_3_A. Based on the analysis of published test results, the ‘S-shape’ curve function is proposed to calibrate the synergistic hydration effect of C_2_S and C_3_S. A series of correction coefficients are introduced into the model to quantify the influence mechanism between each mineral during cement hydration, and the prediction of Portland cement (PC) hydration with the composite model is realized.

### 1.2. Research Significance

Given the existing models’ deficiencies, the predictive hydration models for PC and its main minerals are established in this work. The proposed hydration model is not a pure mathematical regression model but has certain physical significance and predictive ability. Unlike some models with physical background, this model can be applied to the long-term FEA of structures by introducing reasonable assumptions and simplifications. Moreover, this model retains the ability to predict the microstructure evolution of PC, which of course, requires the introduction of real initial microstructure. This work’s main contribution lies in the establishment of the predictive hydration models of the main minerals in PC, and the accurate prediction of PC hydration is realized based on the compound of these models. Most hydration models establish a functional relationship between mineral composition and parameters through statistical analysis of mass data, which is reasonable, but has high requirements on the quantity and quality of data sets. The present composite model here can truly reflect the influence of mineral composition on the hydration of different PC systems, so as to describe the hydration process of PC system more accurately. Furthermore, this model’s compositionality also makes it have the potential to be further extended, such as introducing the hydration mechanisms of new minerals into the model to predict the hydration of modified cement.

This model’s applicability in the long-term FEA of structures guarantees its potential application in civil engineering and concrete technology. By introducing coefficients to further describe the influence of admixtures such as fly ash and slag on the cement hydration, the hydration process and temperature evolution process of structural concrete can be accurately predicted, which is of great significance to the design of temperature control measures for mass concrete. Based on the hydration model, the prediction model of creep, strength, and autogenous volume deformation of coagulation can be further developed in combination with relevant experiments, to predict the long-term mechanical properties evolution of concrete and optimize the design of structures. As mentioned above, the model has a certain application potential in the development of modified cement. Considering the limitation of the physical experiment in scale and duration, it is valuable to establish a hydration model to discuss the application of modified cement in engineering from the perspective of numerical analysis.

## 2. Hydration Mechanism of Cement

Cement hydration involves a collection of complex chemical reactions and physical changes, and its energy conversion rate (mainly in the form of exothermic rate), depends on the reactants and reaction mechanism. PC is the most widely used cement, and its clinker consists of main minerals and secondary minerals.

The main minerals include alite, belite, C_3_A, and tetra-calcium aluminoferrite (C_4_AF), with a mass fraction of about 55%, 20%, 10%, and 8%, respectively. Sub-minerals include free-CaO, periclase and alkali minerals. Calcium silicate accounts for more than 70% of the total cement quality, and makes a major contribution to the cement strength. Numerous studies [19,20,21] indicate that the reactions of C_3_S and C_2_S with water are similar, both producing calcium hydrated silicate (C-S-H) and Ca (OH)_2_. C-S-H is a variable stoichiometric amorphous gel network composed of very fine particles [22]. Despite decades of research on C-S-H, its approximate molecular structure has not been proposed until the development of neutron scattering technology and molecular dynamics simulation in recent years [22,23,24]. The approximate reaction equations for C_3_S and C_2_S can be derived based on the above studies (in the reactions listed here, C = CaO, H = H_2_O, S = SiO_2_, A = Al_2_O_3_, F = Fe_2_O_3_, and S = SO_3_):

(1) $$C_{3}S+3.1H\to C_{1.7}SH_{1.8}+1.3\mathrm{CH}$$

(2) $$C_{2}S+2.1H\to C_{1.7}{\mathrm{SH}}_{1.8}+0.3\mathrm{CH}$$

C_3_A reacts very quickly with water, producing poorly crystalline hydrated calcium aluminate. The calcium aluminate hydrate layer has a fairly penetrable structure and gradually transforms into stable calcium aluminate hydrate crystals as the reaction proceeds. Experiments show that the conversion process will start in a very short time at room temperature [25], and the overall reaction equation is

(3) $$C_{3}A+6H\to C_{3}{\mathrm{AH}}_{6}$$

Gypsum is often added to cement as a retarder. The retarding mechanism is the recation of gypsum and C_3_A to form ettringite (AFt), which is shown in Equation (4). The rate of this reaction is much lower than that of C_3_A directly reacting with water. When gypsum is consumed earlier than C_3_A, ettringite will react with C_3_A to form monosulfur hydrated calcium sulfoaluminate (AFm), as shown in Equation (5)

(4) $$C_{3}A+3C\bar{S}H_{2}+26H\to C_{6}A{\bar{S}}_{3}H_{32}$$

(5) $${2C}_{3}{A+C}_{6}A{\bar{S}}_{3}H_{32}+4H\to {3C}_{4}A\bar{S}H_{12}$$

The reaction mechanism of C_4_AF is similar to that of C_3_A. C_4_AF can react with gypsum to produce ettringite and AFm replaced by iron phase

(6) $$C_{4}\mathrm{AF}+10H\to C_{3}{\mathrm{AH}}_{6}+\mathrm{CH}+\mathrm{FH}3$$

(7) $$C_{4}\mathrm{AF}+3C\bar{S}H_{2}+30H\to C_{6}{A\bar{S}}_{3}H_{32}{+\mathrm{CH}+\mathrm{FH}}_{3}$$

(8) $${2C}_{4}{\mathrm{AF}+C}_{6}A\bar{S_{3}}H_{32}+12H\to 3C_{4}A\bar{S_{3}}H_{12}{+2\mathrm{CH}+2\mathrm{FH}}_{3}$$

The chemical reaction equations listed above are approximate. In fact, the hydration reaction of the main minerals varies with time and depends on the cement. For example, the true reaction of C_3_A-gypsum system is very complex, ettringite, AFm, and C_3_AH_6_ may coexist at some time. In this work, an ideal three-stage reaction sequence is proposed to describe the hydration of C_3_A-gypsum system. Modeling based on approximate reaction equations may lead to differences in the types and quantities of the simulated compounds from the actual ones. However, in view of the complexity and uncertainty of the real cement hydration and the scientific nature of the approximate reaction formula in statistics, it is feasible to adopt the approximate reaction formula for modeling.

As summarized by Bullard et al. [26], cement hydration can be divided into the following main processes:

- Dissolution: ions, such as Ca^2+^ and SiO_4_^4−^, escape from the surface of cement particles in contact with water.
- Adsorption: ions or molecules accumulate at the solid–liquid interface.
- Complexation: each ion forms ion-pair complexes on the solid surface.
- Nucleation: when the volume free energy driving force of the formed solid exceeds the energy barrier, hydration products, such as C-S-H nanospheres, precipitate on the solid surface.
- Growth: self-similar growth of a solid core with a time-varying growth rate.
- Diffusion: the growing solids overlap, causing the surface of the cement particles to be covered by hydrate, and the ions and molecules involved in the reaction are transported through the pores of the cement slurry.

## 3. Calibration of Hydration Reaction

### 3.1. Reactants and Products

According to the reaction equation of the main minerals in cement and the physical properties of the components listed in Table 1, the volume of water consumed by the reaction of the main minerals per unit volume and the volume of corresponding products can be calculated. It is worth noting that the densities of some components in Table 1 are approximate. The use of approximate densities may result in differences between the calculated component volumes and the actual volumes. However, these densities are derived from multiple literatures and are considered to be representative to a certain extent. The calculation based on the reaction Equations (1)–(8) is as follows:

(1) The volume of water consumed by unit volume of C_3_S hydration is composed of the volume of water (w) involved in the reaction $\varsigma_{\mathrm{react}(w-C_{3}S)}$ and the volume of water filling the gel pores in C-S-H $\varsigma_{fgp(w{-C}_{3}S)}$:
  $$\varsigma_{w{-C}_{3}S}=\varsigma_{\mathrm{react}(w{-C}_{3}S)}+\varsigma_{fgp(w{-C}_{3}S)}$$
  $$\varsigma_{\mathrm{react}(w{-C}_{3}S)}=3.1\times 1.80\times 10^{-5}/(7.24\times 10^{-5})=0.771$$

**Figure 1:**
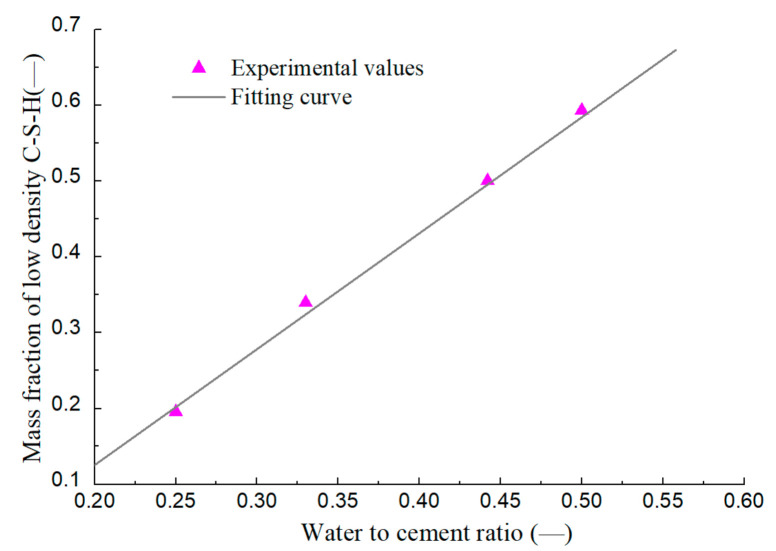
Relationship between mass fraction of low-density C-S-H and w/c of cement sample.

Studies [39,40] have indicated that the cement hydration reaction generates high density C-S-H gel and low-density C-S-H gel. For simplification, the approximate porosity of high-density C-S-H and low-density C-S-H is set as 0.26 and 0.36, respectively, according to [39,41,42]. Tennis’ analysis [41] of Hunt’s test [43] showed the two C-S-H types’ mass ratios changed little when the hydration degree of cement exceeded 0.3. The linear relationship between the mean mass fraction of low-density C-S-H and the water–cement ratio is established through the analysis of the test data, as shown in Figure 1. This approximation simplifies the model, but also increases the dependence of the model on parameters.

Therefore, the porosity of C-S-H gels $\phi_{gp}$ and the saturation of C-S-H gel pores $S_{gp}$ can be estimated with

$$0.26\;\leq \phi_{gp}=0.2425+0.15w/c\leq 0.36$$

$$S_{gp}=0.67+0.33S_{cp}$$

$$\varsigma_{fgp(w{-C}_{3}S)}=\phi_{gp}S_{gp}\varsigma_{{C-S-H-C}_{3}S}$$

$$\varsigma_{{C-S-H-C}_{3}S}=1.10\times 10^{-4}/(7.24\times 10^{-5})=1.519$$

$$\varsigma_{{\mathrm{CH}-C}_{3}S}=1.3\times 3.30\times 10^{-5}/(7.24\times 10^{-5})=0.593$$

(2) The volume of water consumed by C_2_S hydration is also composed of two parts
  $$\varsigma_{w{-C}_{2}S}=\varsigma_{\mathrm{react}(w{-C}_{2}S)}+\varsigma_{fgp(w{-C}_{2}S)}$$
  $$\varsigma_{\mathrm{react}(w{-C}_{2}S)}=2.1\times 1.80\times 10^{-5}/(5.24\times 10^{-5})=0.721$$
  $$\varsigma_{{C-S-H-C}_{2}S}=1.10\times 10^{-4}/(5.24\times 10^{-5})=2.099$$
  $$\varsigma_{{\mathrm{CH}-C}_{2}S}=0.3\times 3.30\times 10^{-5}/(5.24\times 10^{-5})=0.189$$
(3) Since the reaction of C_3_A is divided into three stages, $\varsigma_{w{-C}_{3}A}$, the volume of water consumed by C_3_A hydration, is controlled by the reaction in different stages. In stage I, $\varsigma_{{}_{w{-C}_{3}A}}^{I}$, $\varsigma_{{}_{C\bar{S}H_{2}{-C}_{3}A}}^{I}$ and $\varsigma_{{}_{{\mathrm{Aft}-C}_{3}A}}^{I}$ is calculated as
  $$\varsigma_{{}_{w{-C}_{3}A}}^{I}=26\times 1.80\times 10^{-5}/(8.88\times 10^{-5})=5.270,\;\varsigma_{{}_{C\bar{S}H_{2}{-C}_{3}A}}^{I}=3\times 7.41\times 10^{-5}/(8.88\times 10^{-5})=2.504,\;\varsigma_{{}_{{\mathrm{AFt}-C}_{3}A}}^{I}=7.05\times 10^{-4}/(8.88\times 10^{-5})=7.941$$
  In stage II, $C\bar{S}H_{2}$ has been consumed and C_3_A mainly reacts with ettringite. $\varsigma_{{}_{w{-C}_{3}A}}^{II}$, $\varsigma_{{}_{A{\mathrm{ft}-C}_{3}A}}^{II}$ and $\varsigma_{{}_{A{\mathrm{fm}-C}_{3}A}}^{II}$ is calculated as
  $$\varsigma_{{}_{w{-C}_{3}A}}^{II}=4\times 1.80\times 10^{-5}/(2\times 8.88\times 10^{-5})=0.405,\;\varsigma_{{}_{{\mathrm{AFt}-C}_{3}A}}^{II}=7.05\times 10^{-4}/(2\times 8.88\times 10^{-5})=3.971$$
  $$\varsigma_{{}_{{\mathrm{AFm}-C}_{3}A}}^{II}=3\times 3.09\times 10^{-4}/(2\times 8.88\times 10^{-5})=5.213$$
  In stage III, ettringite is consumed and C_3_A reacts with water directly
  $$\varsigma_{{}_{w{-C}_{3}A}}^{III}=6\times 1.80\times 10^{-5}/(8.88\times 10^{-5})=1.227,\;\varsigma_{{}_{w{-C}_{3}{\mathrm{AH}}_{6}}}^{III}=1.49\times 10^{-4}/(8.88\times 10^{-5})=1.682$$
(4) Similar to C_3_A, the volume change of each component in the C_4_AF hydration can also be calculated in three stages
  $$\varsigma_{{}_{w{-C}_{4}AF}}^{I}=30\times 1.80\times 10^{-5}/(1.29\times 10^{-4})=4.186,\;\varsigma_{{}_{C\bar{S}H_{2}{-C}_{4}\mathrm{AF}}}^{I}=3\times 7.41\times 10^{-5}/(1.29\times 10^{-4})=1.723,$$
  $$\varsigma_{{}_{w{-C}_{4}AF}}^{I}=30\times 1.80\times 10^{-5}/(1.29\times 10^{-4})=4.186,\;\varsigma_{{}_{C\bar{S}H_{2}{-C}_{4}\mathrm{AF}}}^{I}=3\times 7.41\times 10^{-5}/(1.29\times 10^{-4})=1.723,$$
  $$\varsigma_{{}_{\left({\mathrm{AFt}+\mathrm{FH}}_{3}+\mathrm{CH}\right){-C}_{4}\mathrm{AF}}}^{I}=(7.05\times 10^{-4}+7.13\times 10^{-5}+3.30\times 10^{-5})/(1.29\times 10^{-4})=6.273;$$
  $$\varsigma_{{}_{\left({\mathrm{AFm}+\mathrm{FH}}_{3}+\mathrm{CH}\right){-C}_{4}\mathrm{AF}}}^{II}=(3\times 3.09\times 10^{-4}+2\times 7.13\times 10^{-5}+2\times 3.30\times 10^{-5})/(2\times 1.29\times 10^{-4})=3.904$$
  $$\varsigma_{{}_{w{-C}_{4}AF}}^{III}=1.80\times 10^{-5}/1.29\times 10^{-4}=\;1.395,\;\varsigma_{{}_{\left(C_{3}{\mathrm{AH}}_{6}{+\mathrm{FH}}_{3}+\mathrm{CH}\right)-}{}_{C_{4}\mathrm{AF}}}^{III}=(1.49\times 10^{-4}+7.13\times 10^{-5}+3.30\times 10^{-5})/(1.29\times 10^{-4})=1.236$$

### 3.2. Specific Surface Area and Equivalent Particle Size of Particles

The real cement is composed of various particles with different sizes, and its fineness is generally described by the particle size distribution curve or specific surface area. For simplification, the equivalent initial particle radius $r_{eq}^{}$ is introduced. The specific surface area calculated from the particle size distribution curve is equal to that of the particle system with all radius = $r_{eq}^{}$, which can be expressed in Equation (9). In this study, Blaine fineness = 350 kg/m^2^ corresponds to particle size = 13 µm i.e., radius = 6.5 µm.

(9) $$\begin{array}{l} Specific\;surface\;area=\frac{A^{tot}}{V_{0}^{}\rho }=\int {}_{V}\frac{\frac{dV_{0}^{}(r)}{(4/3)\pi r^{3}}4\pi r^{2}}{V_{0}^{}\rho }=\int {}_{V}\frac{3}{r}\frac{dV_{0}^{}(r)}{V_{0}^{}\rho }=\int {}_{V}\frac{3}{r}\frac{dV_{0}^{}(r)}{V_{0}^{}\rho }\\ =\int {}_{r}\frac{3}{r}f(2r)dr=\frac{3}{\rho r_{eq}} \end{array}$$

where f(r) is the mass distribution function of particle size, which is generally described by the following equation, among which p is the fitting coefficient and $r_{avg}$ is the average radius of the particles.

(10) $$f(r)=\frac{p{\left(r/r_{avg}\right)}^{p-1}}{r_{avg}{\left[1+{\left(r/r_{avg}\right)}^{p}\right]}^{2}}$$

## 4. Hydration of C_3_S and Alite

Alite is a kind of unclean polycrystalline of C_3_S with impurities such as ${\mathrm{Al}}_{2}O_{3}$, ${\mathrm{Fe}}_{2}O_{3}$, SrO, and MgO, etc. [44]. Alite is prepared by mixing calcium carbonate, silica, alumina and magnesium oxide in a certain molar ratio. The addition of aluminum and magnesium can promote the nucleation and grain growth of C_3_S in two aspects [45]: (1) A small amount of Mg^2+^ increases the quantity of the liquid phase and reduces the viscosity of the mixture, which is conducive to the diffusion of chemical substances and the growth of grain; (2) Al^3+^ and Mg^2+^ can help to stabilize C_3_S crystals.

### 4.1. Hydration Characteristics of C_3_S and Alite

**Figure 2:**
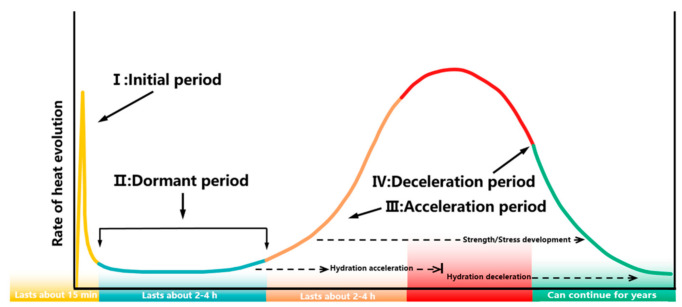
Hydration process of C_3_S and alite.

The hydration of C_3_S and alite can be divided into four periods (Figure 2):

(1) Initial rapid reaction period: C_3_S reacts quickly with water after wetting, verified by the strong exothermic signal in the isothermal calorimetry test. The results of chemical analysis illustrate that the rapid dissolution of C_3_S is also one reason for the strong exothermic signals [46,47].
(2) Dormant period: The theoretical mechanism of this period has always been controversial, and there are many proposed mechanisms, such as metastable barrier hypothesis, surface hydroxylation hypothesis, crystal dissolution hypothesis, lattice defect hypothesis, and C-S-H precipitation hypothesis. The metastable barrier hypothesis and crystal dissolution hypothesis are widely discussed. The metastable barrier hypothesis, proposed by Stein et al. [48] and perfected by Jennings et al. [49] and Mehta [50], suggests that in the initial period, the unhydrated C_3_S surface will gradually form a continuous but thin metastable layer composed of calcium silicate hydrate phase with high Ca/Si, which can effectively passivate the surface by limiting its contact with water, thus reducing the hydration rate of C_3_S. In related studies, CP-MAS NMR technology [51], XPS technology [52], NRRA technology [53,54], and QENS technology [55] were adopted to confirm the possibility of the existence of a protective layer indirectly. However, the lack of direct evidence is still the biggest weakness of this theory. In 2010, Juilland et al. [56] proposed the crystal dissolution hypothesis, which held that the C_3_S unsaturation of pore solution gradually decreased with the reaction, and the C_3_S dissolved in the way of step wave fading with a slow rate. Compared with the metastable barrier hypothesis, the crystal dissolution hypothesis is more verifiable and supported by some experimental phenomena [57,58,59]. Recently, Hu et al. [60] observed the three-dimensional morphology changes of alite during the dormant period through nanometer CT and found no protective layer, only the formation and filling of corrosion pit on the surface of alite was found.
(3) Acceleration reaction period: The hydration rate is controlled by the heterogeneous nucleation and self-similar growth of C-S-H on the C_3_S surface. Reaction-diffusion theory [61], C-S-H gel precipitation control theory [62,63,64], and C_3_S dissolution control theory [65] have been proposed to explain the acceleration period.
(4) Deceleration reaction period: Diffusion control theory is generally considered to be the main reason for the deceleration period. C-S-H forms a complete and continuous barrier on the surface of unhydrated C_3_S, and the hydration reaction is mainly controlled by the inward diffusion of water and the outward diffusion of ions. There are also different opinions. Bishnoi et al. [66] proposed that the deceleration period was controlled by the filling and densification of the C-S-H gel. Bullard et al. [67] and Nicoleau et al. [65] suggested that the deceleration period was due to the reduction of the effective dissolved area of C_3_S.

### 4.2. Hydration Model Based on Water Diffusion Theory

**Figure 3:**
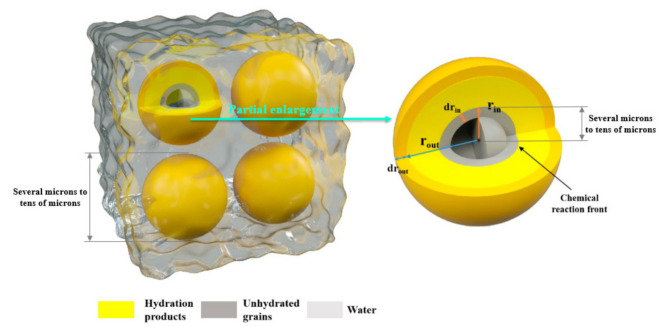
Schematic diagram of particle hydration.

The calculation unit of the model is C_3_S particles, the hydration kinetics of which is controlled by the radial diffusion of water into the porous C-S-H shell. The model has the following important assumptions:

- The decrease of inner radius and the increase of outer radius due to hydration are isotropic (The schematic process of particle hydration is shown in Figure 3);

- The inward diffusion of water through the C-S-H shell is isotropic.

#### 4.2.1. Governing Equation

The inward radial diffusion of water is driven by the chemical potential gradient of pore water. The radial diffusion flow rate Qw(r) of water can be expressed as a function of relative humidity H

(11) $$Qw(r)=4\pi r^{2}B_{eff}\frac{dH(r)}{dr}$$

where, *r* is the radial coordinate; $B_{{}_{eff}}$ is radial effective diffusion coefficient of water passing through C-S-H shell. By integrating Equation (11)

(12) $$H(r)=\frac{-Qw(r)}{4\pi rB_{eff}}+C$$

By substituting $H(r_{in})=H_{c},H(r_{out})=H$ as boundary conditions, the water flow through the C-S-H shell at time *t* can be obtained as

(13) $$Qw_{t}=4\pi r_{in}(t)r_{out}(t)B_{eff}\frac{H(t)-H_{c}}{r_{out}(t)-r_{in}(t)}$$

where, $r_{in}$ is the radius of unhydrated particle; $r_{out}$ is the outside radius of particles after hydration; H is the relative humidity in pores; $H_{c}$ is the relative humidity at the interface between unhydrated particles and C-S-H shell.

Based on desorption isotherm and Fick’s law, the relation between relative humidity and water content can be described by Equation (14)

(14) $$\frac{1}{k_{H}}\frac{\partial H\left(t\right)}{\partial t}+\left(n_{eq}^{}\frac{Qw_{t}}{\phi_{cap}(t)}+\frac{k_{H}+H\left(t\right)-1}{k_{H}\phi_{cap}(t)}\frac{\partial \phi_{cap}(t)}{\partial t}\right)=\nabla \frac{D_{H}}{\rho_{water}}\nabla H$$

where, $\phi_{cap}$ is capillary porosity; $D_{H}$ is moisture permeability; $k_{H}$ is the inverse slope of the desorption isotherm, which can be estimated by the empirical formula shown in Equation (15) suggested by [30]

(15) $$\frac{1}{k_{H}}=m_{2}+(m_{1}-m_{2})\frac{1}{1+{\left(\frac{1-H}{1-H^{*}}\right)}^{2}}$$

(16) $$m_{1}=\frac{\xi_{u}}{\xi }\left[2.4+{\left(5.26w/c-0.68\right)}^{1.5}\right]$$

(17) $$m_{2}=1.18{\left(w/c\right)}^{0.4}$$

(18) $$H^{*}=1-\frac{\xi_{u}}{\xi }\frac{0.03}{{\left(w/c\right)}^{2}}$$

#### 4.2.2. Volume Change of Components during Hydration

Given $Qw_{t}$, the volume change of C_3_S, C-S-H, and CH, the change of inner and outer radius and the change of capillary porosity can be converted

(19) $$dV_{}^{C_{3}S}(t)=-n_{eq}^{C_{3}S}Qw_{t}\frac{1}{\varsigma_{w-C_{3}S}}\exp\left(-\frac{E_{a}}{R}\left(\frac{1}{T}-\frac{1}{273}\right)\right)dt$$

(20) $$dV_{}^{C-S-H^{C_{3}}{}^{S}}(t)=n_{eq}^{C_{3}S}Qw_{t}\frac{\varsigma_{\left(C-S-H-C_{3}S\right)}}{\varsigma_{w-C_{3}S}}\exp\left(-\frac{E_{a}}{R}\left(\frac{1}{T}-\frac{1}{273}\right)\right)dt$$

(21) $$dV_{}^{CH^{C_{3}}{}^{S}}(t)=n_{eq}^{C_{3}S}Qw_{t}\frac{\varsigma_{\left(CH-C_{3}S\right)}}{\varsigma_{w-C_{3}S}}\exp\left(-\frac{E_{a}}{R}\left(\frac{1}{T}-\frac{1}{273}\right)\right)dt$$

(22) $$dr_{in}(t)=-Qw_{t}\frac{1}{\varsigma_{w-C_{3}S}}\frac{1}{4\pi (r_{0}^{eq}{)}^{2}}\exp\left(-\frac{E_{a}}{R}\left(\frac{1}{T}-\frac{1}{273}\right)\right)dt$$

(23) $$dr_{\mathrm{out}}(t)=Qw_{t}\left(\frac{\varsigma_{\left(C-S-H-C_{3}S\right)}}{\varsigma_{w-C_{3}S}}-\frac{1}{\varsigma_{w-C_{3}S}}\right)\frac{1}{S_{out}}\exp\left(-\frac{E_{a}}{R}\left(\frac{1}{T}-\frac{1}{273}\right)\right)dt$$

(24) $$dr_{\mathrm{out}}(t)=Qw_{t}\left(\frac{\varsigma_{\left(C-S-H-C_{3}S\right)}}{\varsigma_{w-C_{3}S}}-\frac{1}{\varsigma_{w-C_{3}S}}\right)\frac{1}{S_{out}}\exp\left(-\frac{E_{a}}{R}\left(\frac{1}{T}-\frac{1}{273}\right)\right)dt$$

(25) $$d\phi_{cap}(t)=-\left(dV_{}^{C-S-H^{C_{3}}{}^{S}}+dV_{}^{CH^{C_{3}}{}^{S}}+dV_{}^{C_{3}S}\right)+\left(\phi_{gp}-\phi_{np}\right)dV_{}^{C-S-H^{C_{3}}{}^{S}}$$

where, $n_{}^{eq}$ is the number of equivalent particles; *T* is the Kelvin temperature; *R* is the ideal gas constant; $S_{out}$ is the remaining area of the particle surface after removing the overlapping area between particles, and its calculation method will be introduced in the next section.

#### 4.2.3. Hydration Rate of C_3_S and Alite

The hydration rate of C_3_S and alite can be obtained by definition

(26) $$\frac{d\xi^{alite/C_{3}S}(t)}{\mathrm{dt}}=\frac{d\left(\frac{V_{0}^{{}^{C_{3}S}}-V_{}^{{}^{C_{3}S}}\left(t\right)}{V_{0}^{{}^{C_{3}S}}}\right)}{\mathrm{dt}}=Qw_{t}\frac{1}{\varsigma_{w-C_{3}S}}\frac{3}{4\pi (r_{}^{eq}{)}^{3}}\exp\left(-\frac{E_{{}_{a}}^{alite/C_{3}S}}{R}\left(\frac{1}{T}-\frac{1}{273}\right)\right)efs\left(r_{out}\right)$$

where, $E_{{}_{a}}^{alite/C_{3}S}$ is the activation energy of C_3_S and alite, which can be set to 46 kJ/mol [68]; $efs\left(r_{out}\right)$ represents the influence of particle surface contact on hydration rate, which can be solved by

(27) $$efs\left(r_{out}\right)=\frac{S_{\mathrm{out}}}{4\pi r_{out}{}^{2}}=\left\{\begin{array}{cc} 1 & r_{out}\leq L_{h}/2\\ \frac{6\pi r_{out}L_{h}-8\pi r_{out}{}^{2}}{4\pi r_{out}{}^{2}} & L_{h}/2<r_{out}\leq \sqrt{2}L_{h}/2\\ \frac{6\pi r_{out}L_{h}+12r_{out}\sqrt{r_{out}{}^{2}-\frac{L_{h}{}^{2}}{2}}+6\pi r_{out}L_{h}\arccos\left(\frac{L_{h}}{\sqrt{2}r_{out}}\right)-8\pi r_{out}{}^{2}}{4\pi r_{out}{}^{2}} & \sqrt{2}L_{h}/2<r_{out}\leq \sqrt{3}L_{h}/2 \end{array}\right.$$

where, $L_{h}$ is the length of hydration space composed of a single particle and water, which can be obtained according to water cement ratio and particle density.

Hydration rates of C_3_S and alite are described by the radial effective diffusion coefficient $B_{eff}$ of water through the C-S-H shell

(28) $$B_{eff}=B_{0}f_{\xi }\left(\xi \right)\frac{1}{1+{\left(\left(1-H\left(t\right)\right)/0.12\right)}^{8}}$$

where $B_{0}$ is a constant value; $f_{\xi }\left(\xi \right)$ is the influence factor of hydration degree on effective diffusion coefficient.

In literature [18], the start point of calculation was the end of dormant period. To simulate the initial period and dormant period, a modified $f_{\xi }\left(\xi \right)$ is proposed here. The modified $f_{\xi }\left(\xi \right)$ describes the hydration process in three stages. The first stage includes the initial period and the dormant period, and the increase of hydration rate marks the end of this stage. In Equation (29), $\xi_{dor}$ represents the hydration degree at the end of the dormant period. When the hydration degree exceeds $\xi_{dor}$, $f_{\xi }\left(\xi \right)$ begins to increase. The remaining two stages describe the acceleration and deceleration period. $\xi_{c}$ is the hydration degree corresponding to the formation of a complete shell of C-S-H. $\xi^{*}$ that distinguishes the second and third stages is empirical and can be calculated from $\xi^{*}=l\xi_{c}$

(29) $$f_{\xi }\left(\xi \right)=\left\{\begin{array}{ccc} {\left(\frac{r_{{}_{eq}}^{}}{r_{{}_{eq}}^{0}}\right)}^{2}{\left(0.35\frac{\left(\xi -\xi_{dor}\right)}{\xi_{dor}}\right)}^{c}+{\left(\frac{2\xi_{dor}}{\xi_{c}}\right)}^{m}e^{-{\left(\frac{2\xi_{dor}}{\xi_{c}}\right)}^{m}} & \mathrm{for} & \xi \leq \xi_{dor}\\ {\left(\frac{2\xi }{\xi_{c}}\right)}^{m}e^{-{\left(\frac{2\xi }{\xi_{c}}\right)}^{m}} & \mathrm{for} & \xi_{dor}<\xi \leq \xi^{*}\\ {\left(\frac{\xi -\xi^{*}+2s\xi^{*}/\xi_{c}\;}{s}\right)}^{m}e^{-{\left(\frac{\xi -\xi^{*}+2s\xi^{*}/\xi_{c}\;}{s}\right)}^{m}} & \mathrm{for} & \xi^{*}<\xi \end{array}\right.$$

(30) $$\xi_{c}=\xi_{{}_{c}}^{0}\frac{r_{{}_{eq}}^{0}}{r_{eq}}\left(1+\frac{1.2}{\xi_{{}_{c}}^{0}}\left(w/c-0.4\right)\right)\exp\left[\frac{E*}{R}\left(\frac{1}{293.15}-\frac{1}{273.15+T}\right)\right]$$

(31) $$\frac{\xi_{c}}{\xi_{{}_{c}}^{0}}=\frac{\xi_{dor}}{\xi_{dor}^{0}}$$

where *c, m, s*, and *l* are empirical parameters; $r_{{}_{eq}}^{0}$ is reference equivalent particle radius, which is 6 µm; $\xi_{{}_{c}}^{0}$ and $\xi_{dor}^{0}$ are the reference values corresponding to $r_{{}_{eq}}^{0}$; $E^{*}/R=800$.

### 4.3. Model Validation

**Figure 4:**
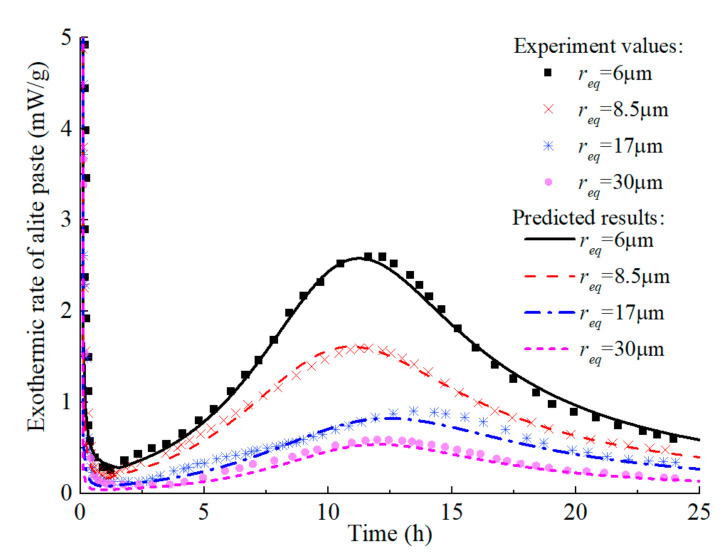
Isothermal calorimetric test values and prediction curves of alite in early age.

**Figure 5:**
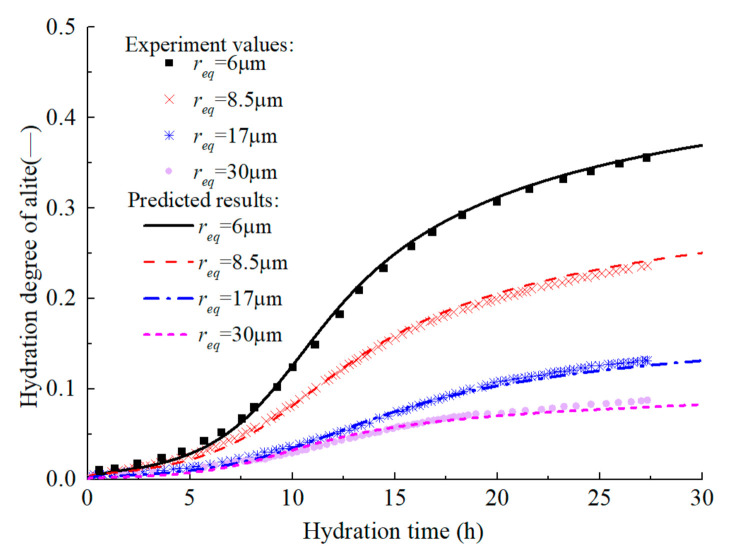
Experimental values and prediction curves of hydration degree of alite in early age.

Based on the above model, the experiment of Costoya [69] is simulated to predict the hydration heat release process of alite and C_3_S in the isothermal calorimetry test. The w/c of each sample in the test was 0.4. For alite, $\xi_{dor}^{0}$ = 0.011 and $\xi_{c}^{0}$ = 0.41 are the recommended values in literature [18]. The model parameters *B_0_* = 1.27 × 10^−16^ m^2^/s, *c* = 3, *m =* 2, *s =* 0.7, and *l =* 0.93 are calibrated using the long-term hydration degree of C_3_S with $r_{{}_{eq}}^{}$ = 30 µm and the initial hydration degree of C_3_S with $r_{{}_{eq}}^{}$ = 6 µm, and other curves are predicted results. Figure 4 demonstrates the isothermal calorimetry test results of alite with different particle fineness in early age. It can be seen that the model can predict the heat release rate of alite with different particle sizes in early age, and accurately describe the four periods of alite hydration, namely the initial hydration period, the dormant period, the accelerated reaction period and the decelerated reaction period. Figure 5 shows the predicted evolution of the early-age hydration degree of alite, which further confirms the model’s accuracy.

**Figure 6:**
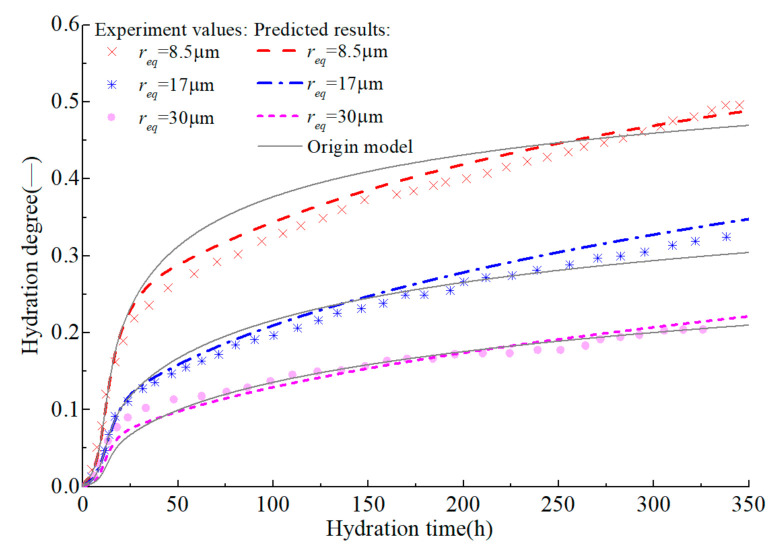
Test values and prediction curves of hydration degree of alite in middle age.

**Figure 7:**
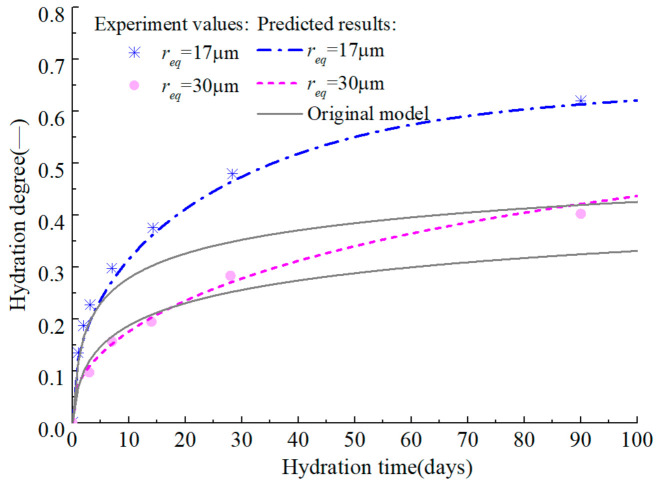
Test values and prediction curves of long-term hydration degree of alite.

On the premise of accurately predicting the early hydration of alite, the development of hydration degree in the medium and long term is further predicted (Figure 6 and Figure 7). The original model [18] can accurately predict the development of the hydration degree of alite in early age, but there is a certain error in the prediction of the middle-term hydration degree, and the error is further expanded in the prediction of the long-term hydration degree. By introducing $efs\left(r_{out}\right)$ which describes the effect of particle surface contact on hydration rate, and modifying the empirical parameters *s* and *l*, the modified model proposed here can predict the hydration degree development of alite at full age.

**Figure 8:**
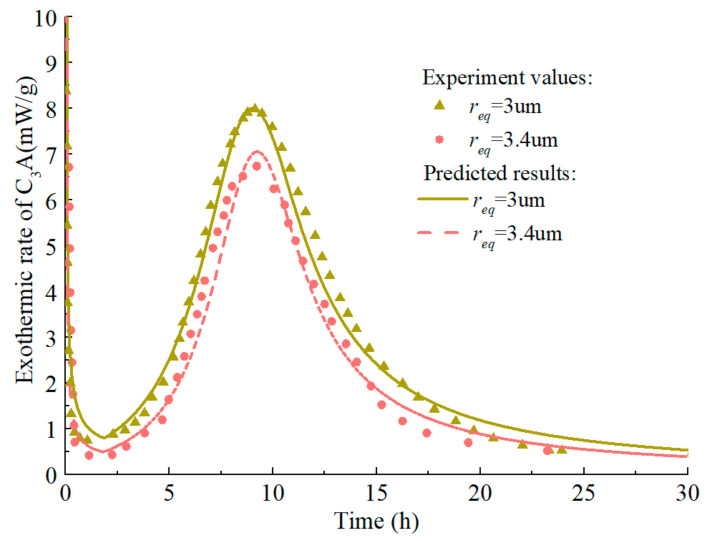
Isothermal calorimetry test values and prediction curves of pure C_3_S in early age.

**Figure 9:**
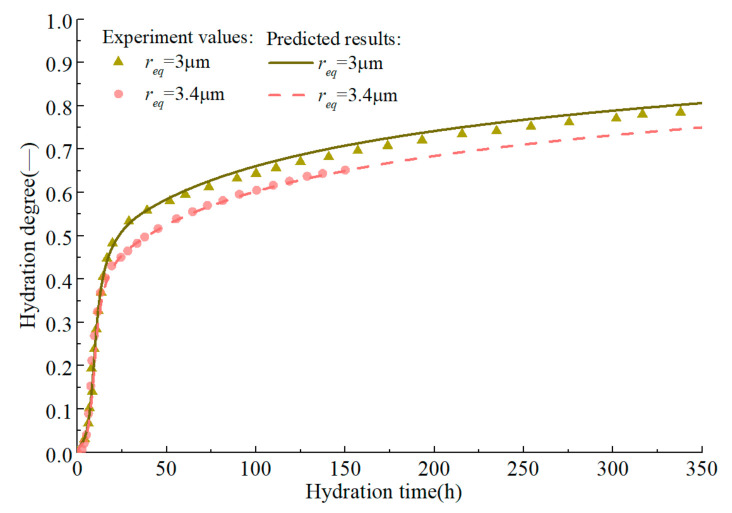
Experimental values and prediction curves of hydration degree of pure C_3_S in middle age.

As mentioned before, the crystalline state of pure C_3_S is not as complete as that of alite. Although the hydration laws of pure C_3_S and alite are consistent, their hydration rates are somewhat different. The parameters of pure C_3_S model are consistent with alite except *B_0_* = 1.04 × 10^−16^ m^2^/s, *m =* 2.2 and ($\xi_{c,C_{3}S}^{0}$,$\xi_{dor,C_{3}S}^{0}$) = 0.7($\xi_{c,alite}^{0}$,$\xi_{dor,alite}^{0}$). As shown in Figure 8 and Figure 9, the initial exothermic rate of pure C_3_S predicted by the model is basically consistent with the measured rate, and the predicted hydration degree of pure C_3_S in middle age is also close to the experimental value.

## 5. Hydration of C_2_S

The hydration of C_2_S, which is the second-highest mineral in PC, has been extensively studied [70,71,72,73]. The hydration mechanism of C_2_S is similar to that of C_3_S, and its hydration reaction can also be divided into initial rapid reaction period, dormant period, accelerated reaction period, and decelerated reaction period, but its hydration activity is much lower than that of C_3_S. The hydration degree of C_2_S in the early age is much lower than that of C_3_S, but in the middle and late age, the hydration rate of C_2_S will increase. The modified hydration model of C_3_S is proposed and verified in the previous section. On this basis, $f_{{}_{\xi }}^{C_{2}S}\left(\xi \right)$ suitable for C_2_S is designed to simulate the hydration characteristics of C_2_S:

(32) $$f_{{}_{\xi }}^{C_{2}S}\left(\xi \right)=\left\{\begin{array}{ccc} {\left(0.35\frac{\left(\xi -\xi_{dor}\right)}{\xi_{dor}}\right)}^{c}+{\left(\frac{2\xi_{dor}}{\xi_{c}}\right)}^{m}e^{-{\left(\frac{2\xi_{dor}}{\xi_{c}}\right)}^{m}} & \mathrm{for} & \xi \leq \xi_{dor}\\ {\left(\frac{2\xi }{\xi_{c}}\right)}^{m}e^{-{\left(\frac{2\xi }{\xi_{c}}\right)}^{m}} & \mathrm{for} & \xi_{dor}<\xi \leq \xi^{*}\\ \frac{1-\xi }{1-\xi^{*}}{\left(\frac{2\xi^{*}}{\xi_{c}}\right)}^{m}e^{-{\left(\frac{2\xi^{*}}{\xi_{c}}\right)}^{m}}+{\left(\frac{2\left(\xi -\xi^{*}\right)}{\xi_{c2}-\xi^{*}}\right)}^{m}e^{-{\left(\frac{2\left(\xi -\xi^{*}\right)}{\xi_{c2}-\xi^{*}}\right)}^{m}} & \mathrm{for} & \xi^{*}<\xi \end{array}\right.$$

(33) $$\frac{\xi_{c}}{\xi_{{}_{c}}^{0}}=\frac{\xi_{c2}}{\xi_{{}_{c2}}^{0}}=\frac{\xi_{dor}}{\xi_{dor}^{0}}$$

where, $\xi_{c2}$ is the hydration degree corresponding to the end of the second hydration peaks in $f_{{}_{\xi }}^{C_{2}S}\left(\xi \right)$; the meanings of the remaining parameters are consistent with those of the previous model.

**Figure 10:**
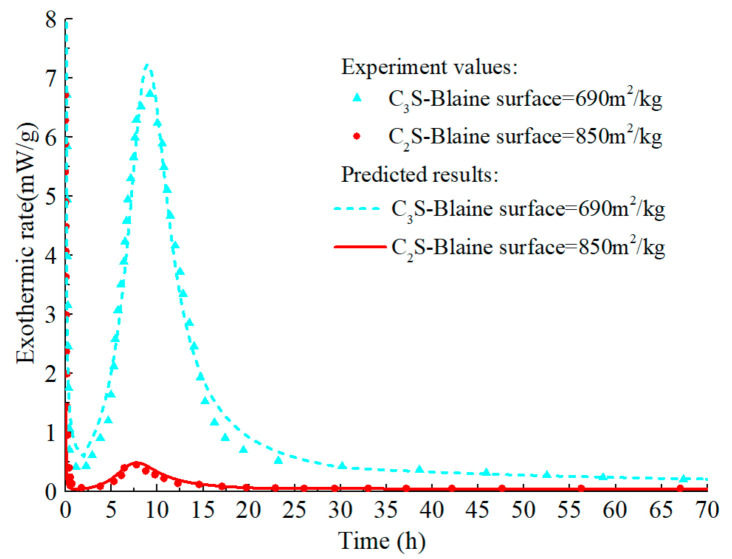
Isothermal calorimetry test values and prediction curves for early hydration of C_2_S and C_3_S.

**Figure 11:**
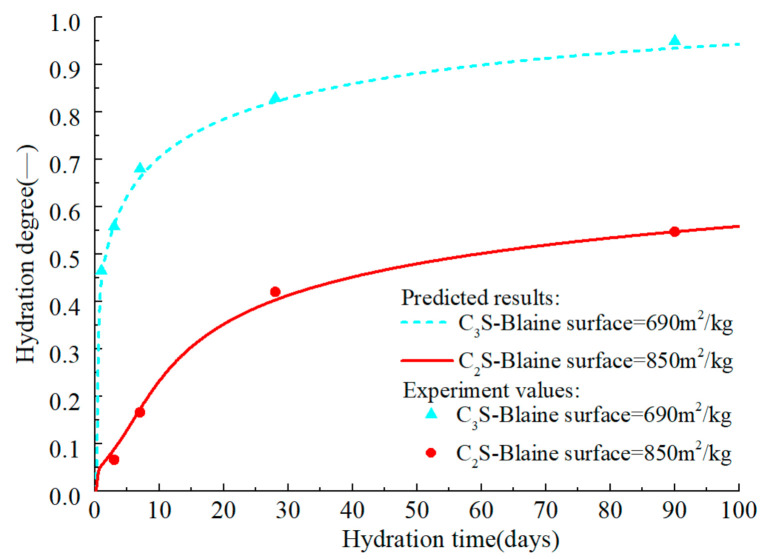
Measured values and prediction curves of long-term hydration degree of C_2_S and C_3_S.

In the hydration model of C_2_S, $B_{0,C_{2}S}$ = 1.27 × 10^−18^ m^2^/s, $\xi_{dor,C_{2}S}^{0}$ = 1.24 × 10^−3^, $\xi_{c,C_{2}S}^{0}$ = 0.025, and $\xi_{c2,C_{2}S}^{0}$ = 0.23 respectively, and the remaining parameters are consistent with those of C_3_S. $E_{a}^{C_{2}S}$ can be set to 32kJ/mol [74]. The model is calibrated by simulating the experiment of Hernández [75], in which the water–cement ratio of all sample was 0.4. As shown in Figure 10 and Figure 11, the model can accurately predict the hydration process of C_2_S and describe the characteristics of the increased reaction rate of C_2_S in the middle and late ages, which is in accordance with the test results. Moreover, the calorimetric test of C_3_S in literature [75] is predicted, which further verifies the C_3_S hydration model’s accuracy. As mentioned above, the hydration mechanism and hydration products of C_2_S and C_3_S are similar, and the impurities in belite and alite are basically the same. Therefore, it is assumed that the relationship between belite and C_2_S is consistent with that between alite and C_3_S.

However, relevant studies have demonstrated that there is a certain synergistic hydration effect in the mixed system of C_2_S and C_3_S. By using quantitative X-ray diffraction analysis, it was concluded that C_3_S can significantly accelerate the hydration rate of C_2_S at any mass ratio of C_2_S to C_3_S, and the hydration rate of C_3_S was inhibited only when a large amount of C_2_S exists in the mixture [76]. Tong and Young [77] also confirmed Odler’s conclusions, and proposed that the accelerated hydration of C_2_S is due to the rapid crystallization of calcium hydroxide in the mixture. Peterson [78] found that in a mixed system with a high C_3_S mass fraction (C_3_S wt % ≥ 80%), the hydration rate of C_3_S increased slightly, and attributed this to the fact that the less reactive C_2_S provided additional nucleation sites for the hydration of C_3_S. Hernandez et al. [75] analyzed the calorimetric data, TGA measurement data, and SI-MAS NUCLEAR magnetic resonance results of the mixed system, and confirmed that C_3_S significantly increases the hydration activity of C_2_S in early age to avoid the inactive dormant period.

In order to quantify the synergistic hydration effect of the C_2_S–C_3_S mixed system, the relationship between the hydration degree of C_2_S and that of C_3_S in the mixed system should be established. For simplification, the following two hypotheses are proposed:

- In the mixed system, only the significant acceleration effect of C_3_S on the hydration of C_2_S is considered, while the weak accelerating effect of C_2_S on C_3_S hydration is ignored.
- The hydration degree of C_3_S in mixed systems is consistent with that of pure C_3_S under the same conditions.

**Figure 12:**
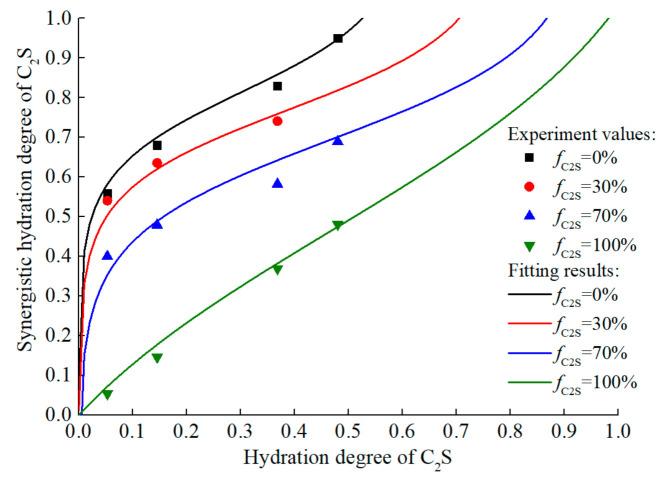
Synergistic hydration effect of mixed systems with different $f_{C_{2}S}^{}$

By analyzing the published test data [75], ‘S’-shaped curve is selected to describe the synergistic hydration effect of C_2_S and C_3_S, and the form and parameters of the function are calibrated, which could describe the response of the system’s synergistic effect to the content of C_2_S. The fitting results were shown in Figure 12

(34) $$\xi_{C_{2}S}^{synergistic}=\frac{\ln\left(\frac{\gamma_{1}\gamma_{2}-\xi_{C_{2}S}^{}\left(1+\gamma_{2}\right)}{\left(1+\gamma_{2}\right)\gamma_{2}\xi_{C_{2}S}^{}+\gamma_{1}\gamma_{2}}\right)}{\gamma_{3}}$$

(35) $$\gamma_{1}=0.7448f_{C_{2}S}+0.5626$$

(36) $$\gamma_{2}=3107.1{\left(f_{C_{2}S}\right)}^{2}-\;6671.8f_{C_{2}S}\;+\;3529.8$$

(37) $$\gamma_{3}=-10e^{-{\left(f_{C_{2}S}\right)}^{4.1}}$$

where, $\xi_{C_{2}S}^{synergistic}$ is hydration degree of C_2_S in mixed system; $f_{C_{2}S}^{}$ is mass percent of C_2_S in mixed system; $\gamma_{1}$, $\gamma_{2}$, and $\gamma_{3}$ are model coefficients.

.

## 6. Hydration of C_3_A

### 6.1. Hydration Properties of C_3_A

**Figure 13:**
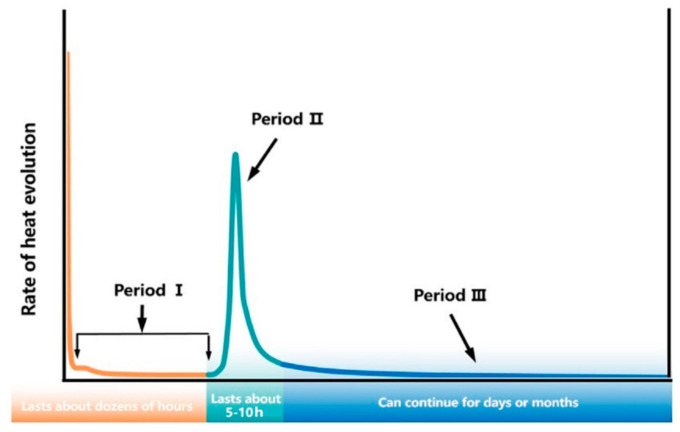
Schematic diagram of C_3_A hydration process.

Generally, gypsum is added as a moderator to avoid the rapid setting of cement caused by the hydration of C_3_A. According to the results of the calorimetric test, the reaction of C_3_A can be divided into three periods with the presence of gypsum, as shown in Figure 13:

(1) The first period corresponds to the reaction between C_3_A and gypsum: in calorimetric tests, period I is characterized by an initial dissolution peak followed by a low heat release phase, which lasts until the gypsum in the system is depleted. The reason for the low heat release rate has been controversial. Some studies, initially, believed that ettringite formed by the reaction between C_3_A and gypsum would be deposited on the surface of C_3_A as a barrier layer to prevent the further hydration of C_3_A [79]. However, Mehta [80] found ettringite was formed by solution mechanism through electron microscopy. Quennoz [81] also observed that hydration rates depend on the specific surface area of C_3_A, further negating the barrier layer theory. The current theory with high acceptability is that Ca^2+^ and SO_4_^2−^ can adsorbed on the surface of C_3_A to form calcium sulfate complex, thus reducing the available dissolution sites on the surface of C_3_A by blocking coordination [26,82]. Recent studies also suggest that the adsorption of calcium sulfate complex on the surface will increase the local saturation of C_3_A and delay the dissolution of C_3_A [83].
(2) The second period corresponds primarily to the reaction of C_3_A and ettringite, and may also include the direct reaction of C_3_A with water: Pommersheim et al. [84] believed that the increase of the reaction rate was due to the removal of the barrier layer by recrystallization of C_3_A and ettringite, while the deceleration period was due to the formation of new barrier layer by the AFm phase. Obviously, this theory is inaccurate in describing the barrier layer of ettringite. Minard et al. [85] pointed out the characteristic shape of hydration peak did not conform to the dissolution control mechanism, and the increase of gypsum content will lead to the broadening of an exothermic peak, which may be caused by the nucleation and growth of more AFm phase, similar to the hydration characteristics of C_3_S [66]. Further research is needed to better understand the second period of C_3_A hydration.
(3) The last period is characterized by a low heat release rate: this period is controlled by a continuation of the previous hydration mechanism, either by dissolution or by nucleation and growth, until the reactant is exhausted.

### 6.2. Hydration Model Based on Dissolution-Water Diffusion Theory

#### 6.2.1. Governing Equation

By analyzing the hydration mechanism of C_3_A, it is believed that the reaction of C_3_A is controlled by the dissolution mechanism in the first period, and assuming the barrier layer theory controls the reaction of C_3_A in the remaining periods, a dissolution-diffusion hydration model of C_3_A is established. In the hydration models of C_3_S and C_2_S, the volume of water consumed by the reaction is calculated first, and then the volume change involved in the reaction is converted. Considering that the volume of water combined with unit volume of C_3_A is different in each stage, the volume reaction rate of C_3_A is used to replace the diffusion flow rate of water to ensure the continuity of the model

(38) $$\frac{dV_{}^{C_{3}A}(t)}{dt}=-n_{eq}^{C_{3}A}4\pi r_{in}(t)r_{out}(t)B_{C_{3}A}\frac{H(t)-H_{c}}{r_{out}(t)-r_{in}(t)}\exp\left(-\frac{E_{{}_{a}}^{C_{3}A}}{R}\left(\frac{1}{T}-\frac{1}{273}\right)\right)efs\left(r_{out}\right)$$

where, $B_{C_{3}A}$ is the effective reaction coefficient of C_3_A; $E_{a}^{C_{3}A}$ represents the reaction activation energy of C_3_A. In the three periods, $E_{a}^{C_{3}A}$ can be taken as 75 kJ/mol, 69 kJ/mol, and 25 kJ/mol in sequence [86].

In the first stage, the reaction mechanism is controlled by dissolution, so the following relation can be deduced

(39) $$r_{out}(t)=r_{\mathrm{in}}^{}(t)\mathrm{when}\xi^{C_{3}A}\leq \xi_{{}^{\mathrm{Gypsum}}}^{C_{3}A}$$

Then, the volume reaction rate of C_3_A in the first stage can be expressed as

(40) $$\frac{dV_{}^{C_{3}A}(t)}{dt}=-n_{eq}^{C_{3}A}4\pi {\left(r_{in}(t)\right)}^{2}C_{C_{3}A}\exp\left(-\frac{E_{{}_{a}}^{C_{3}A}}{R}\left(\frac{1}{T}-\frac{1}{273}\right)\right)efs\left(r_{out}\right)\mathrm{when}\xi^{C_{3}A}\leq \xi_{{}^{\mathrm{Gypsum}}}^{C_{3}A}$$

(41) $$B_{C_{3}A}\frac{H(t)-H_{c}}{r_{in}^{*}(t)-r_{in}(t)}=C_{C_{3}A}$$

where, $C_{C_{3}A}$ is the radial dissolution rate of C_3_A; $\xi_{{}^{\mathrm{Gypsum}}}^{C_{3}A}$ is the hydration degree of C_3_A corresponding to the complete reaction of gypsum; $r_{in}^{*}(t)$ equal $r_{in}(t)$ + Δ$r_{eq}$, and Δ is set to 0.001 in this paper. The purpose of setting $r_{in}^{*}(t)$ is to ensure the computability of the model in the first stage and to ensure the continuity of the model.

In the next two stages, the reaction mechanism of C_3_A is described by barrier theory, so the diffusion model shown in Equation (38) can be used directly as

(42) $$\frac{dV_{}^{C_{3}A}(t)}{dt}=-n_{eq}^{C_{3}A}4\pi r_{in}(t)r_{out}(t)B_{C_{3}A}\frac{H(t)-H_{c}}{r_{out}(t)-r_{in}(t)}\exp\left(-\frac{E_{{}_{a}}^{C_{3}A}}{R}\left(\frac{1}{T}-\frac{1}{273}\right)\right)efs\left(r_{out}\right)\mathrm{when}\xi^{C_{3}A}>\xi_{{}^{\mathrm{Gypsum}}}^{C_{3}A}$$

#### 6.2.2. Volume Changes of Components during Hydration

After obtaining the volume change of C_3_A at time t, the volume change of water at time t can be calculated according to the chemical reaction

(43) $$dV_{}^{w}(t)=\left\{\begin{array}{cc} \varsigma_{{}_{w-C_{3}A}}^{I}dV_{}^{C_{3}A}(t) & \xi^{C_{3}A}\leq \xi_{{}^{\mathrm{Gypsum}}}^{C_{3}A}\\ \varsigma_{{}_{w-C_{3}A}}^{II}dV_{}^{C_{3}A}(t) & \xi_{{}^{\mathrm{Gypsum}}}^{C_{3}A}<\xi^{C_{3}A}\leq \xi_{{}^{\mathrm{Aft}}}^{C_{3}A}\\ \varsigma_{{}_{w-C_{3}A}}^{III}dV_{}^{C_{3}A}(t) & \xi^{C_{3}A}>\xi_{{}^{\mathrm{Aft}}}^{C_{3}A} \end{array}\right.$$

where, $\xi_{{}^{\mathrm{Aft}}}^{C_{3}A}$ represents the hydration degree of C_3_A when either ettringite or C_3_A is consumed.

In the reaction between C_3_A and gypsum, the volume changes of gypsum and ettringite are

(44) $$dV_{I}^{C\bar{S}H_{2}}(t)=\varsigma_{{}_{C\bar{S}H_{2}-C_{3}A}}^{I}dV_{}^{C_{3}A}(t)\xi^{C_{3}A}\leq \xi_{{}^{\mathrm{Gypsum}}}^{C_{3}A}$$

(45) $$dV_{I}^{A\mathrm{ft}}(t)=-\varsigma_{{}_{A\mathrm{ft}-C_{3}A}}^{I}dV_{}^{C_{3}A}(t)\xi^{C_{3}A}\leq \xi_{{}^{\mathrm{Gypsum}}}^{C_{3}A}$$

In the reaction between C_3_A and ettringite, the volume changes of ettringite and AFm are

(46) $$dV_{II}^{A\mathrm{ft}}(t)=\varsigma_{{}_{A\mathrm{ft}-C_{3}A}}^{II}dV_{}^{C_{3}A}(t)\xi_{{}^{\mathrm{Gypsum}}}^{C_{3}A}<\xi^{C_{3}A}\leq \xi_{\mathrm{Aft}}^{C_{3}A}$$

(47) $$dV_{II}^{A\mathrm{fm}}(t)=-\varsigma_{{}_{A\mathrm{fm}-C_{3}A}}^{II}dV_{}^{C_{3}A}(t)\xi_{{}^{\mathrm{Gypsum}}}^{C_{3}A}<\xi^{C_{3}A}\leq \xi_{\mathrm{Aft}}^{C_{3}A}$$

In the direct reaction of C_3_A with water, the volume change of C_3_AH_6_ is

(48) $$dV_{III}^{C_{3}AH_{6}}(t)=-\varsigma_{{}_{w-C_{3}AH_{6}}}^{III}dV_{}^{C_{3}A}(t)\xi^{C_{3}A}>\xi_{{}^{\mathrm{Aft}}}^{C_{3}A}$$

The changes of capillary porosity, inner radius, and outer radius are

(49) $$d\phi_{cap}(t)=\left\{\begin{array}{cc} -\left(dV_{I}^{C\bar{S}H_{2}}+dV_{I}^{A\mathrm{ft}}+dV_{}^{C_{3}A}\right) & \xi^{C_{3}A}\leq \xi_{{}^{\mathrm{Gypsum}}}^{C_{3}A}\\ -\left(dV_{II}^{A\mathrm{ft}}+dV_{II}^{A\mathrm{fm}}+dV_{}^{C_{3}A}\right) & \xi_{{}^{\mathrm{Gypsum}}}^{C_{3}A}<\xi^{C_{3}A}\leq \xi_{\mathrm{Aft}}^{C_{3}A}\\ -\left(dV_{III}^{C_{3}AH_{6}}+dV_{}^{C_{3}A}\right) & \xi^{C_{3}A}>\xi_{{}^{\mathrm{Aft}}}^{C_{3}A} \end{array}\right.$$

(50) $$dr_{in}(t)=\frac{dV_{}^{C_{3}A}(t)}{n_{eq}^{C_{3}A}4\pi (r_{}^{eq}{)}^{2}}$$

(51) $$dr_{\mathrm{out}}(t)=\left\{\begin{array}{cc} dr_{\mathrm{in}}(t) & \xi^{C_{3}A}\leq \xi_{{}^{\mathrm{Gypsum}}}^{C_{3}A}\\ \frac{dV_{II}^{A\mathrm{ft}}+dV_{II}^{A\mathrm{fm}}+dV_{}^{C_{3}A}}{n_{eq}^{C_{3}A}S_{out}} & \xi_{{}^{\mathrm{Gypsum}}}^{C_{3}A}<\xi^{C_{3}A}\leq \xi_{\mathrm{Aft}}^{C_{3}A}\\ \frac{dV_{III}^{C_{3}AH_{6}}+dV_{}^{C_{3}A}}{n_{eq}^{C_{3}A}S_{out}} & \xi^{C_{3}A}>\xi_{{}^{\mathrm{Aft}}}^{C_{3}A} \end{array}\right.$$

#### 6.2.3. Hydration Rate of C_3_A

Given the mass percentage of gypsum $f_{{}_{gypsum}}^{C_{3}A}$ and the molar mass *M* of each component, the following equation can be used to calculate $\xi_{{}^{\mathrm{Gypsum}}}^{C_{3}A}$ and $\xi_{\mathrm{Aft}}^{C_{3}A}$ respectively:

(52) $$\xi_{{}^{\mathrm{Gypsum}}}^{C_{3}A}=\frac{f_{{}_{gypsum}}^{C_{3}A}M_{C_{3}A}}{3M_{gypsum}\left(1-f_{{}_{gypsum}}^{C_{3}A}\right)}$$

(53) $$\xi_{\mathrm{Aft}}^{C_{3}A}=\min\left(\frac{f_{{}_{gypsum}}^{C_{3}A}M_{C_{3}A}}{M_{gypsum}\left(1-f_{{}_{gypsum}}^{C_{3}A}\right)},0\right)$$

The form of $B_{C_{3}A}$ is consistent with that of $B_{eff}$, and $f_{\xi }^{C_{3}A}\left(\xi \right)$ applicable to C_3_A is proposed here as

(54) $$f_{\xi }^{C_{3}A}\left(\xi \right)=\left\{\begin{array}{ccc} \begin{array}{c} \frac{r_{{}_{eq}}^{}}{r_{{}_{eq}}^{0}}{\left(\max\left(1-10\xi ,0\right)\right)}^{c}+\theta_{g}^{1} \end{array} & \xi^{C_{3}A}\leq \xi_{{}^{Gypsum}}^{C_{3}A} & \\ \theta_{g}^{1}+\theta_{g}^{2}{\left(\frac{\xi -\xi_{{}^{Gypsum}}^{C_{3}A}}{0.24}\right)}^{m}e^{-{\left(\frac{\xi -\xi_{{}^{Gypsum}}^{C_{3}A}}{0.24}\right)}^{m}} & \xi_{{}^{Gypsum}}^{C_{3}A}\xi^{C_{3}A}\leq \xi_{Aft}^{C_{3}A} & \\ \theta_{g}^{1}+\theta_{g}^{2}{\left(\frac{\xi -\xi_{Aft}^{C_{3}A}+s\left(\xi_{Aft}^{C_{3}A}-\xi_{{}^{Gypsum}}^{C_{3}A}\right)/0.24\;}{s}\right)}^{m}e^{-{\left(\frac{\xi -\xi_{Aft}^{C_{3}A}+s\left(\xi_{Aft}^{C_{3}A}-\xi_{{}^{Gypsum}}^{C_{3}A}\right)/0.24\;}{s}\right)}^{m}} & \xi^{C_{3}A}\xi_{{}^{Aft}}^{C_{3}A} & \end{array}\right.$$

(55) $$\theta_{g}^{1}=0.0081\frac{r_{{}_{eq}}^{}}{r_{{}_{eq}}^{0}}\left(1-f_{{}_{gypsum}}^{C_{3}A}\right)$$

(56) $$\theta_{g}^{2}=23.623\frac{r_{{}_{eq}}^{}}{r_{{}_{eq}}^{0}}{\left(f_{{}_{gypsum}}^{C_{3}A}\right)}^{-1.827}$$

where, $\theta_{g}^{1}$ and $\theta_{g}^{2}$ are the coefficients related to $f_{\xi }^{C_{3}A}\left(\xi \right)$ and the equivalent particle size; *c, m*, and *s* are empirical parameters.

### 6.3. Model Validation

The parameters of C_3_A hydration model are set as *B*_0_ = 7.937 × 10^−18^ m^2^/s, *c* = 8, *m =* 1.9, and *s =* 0.3. The isothermal calorimetry test of C_3_A in literature [86] is predicted to verify the accuracy of the model. Two kinds of C_3_A samples with different fineness were used in this experiment. According to the particle size distribution curve, the equivalent radius $r_{{}_{eq}}^{fine}$ of fine particles and $r_{{}_{eq}}^{coarse}$ of coarse particles are estimated to be 2.1 µm and 8.2 µm respectively.

**Figure 14:**
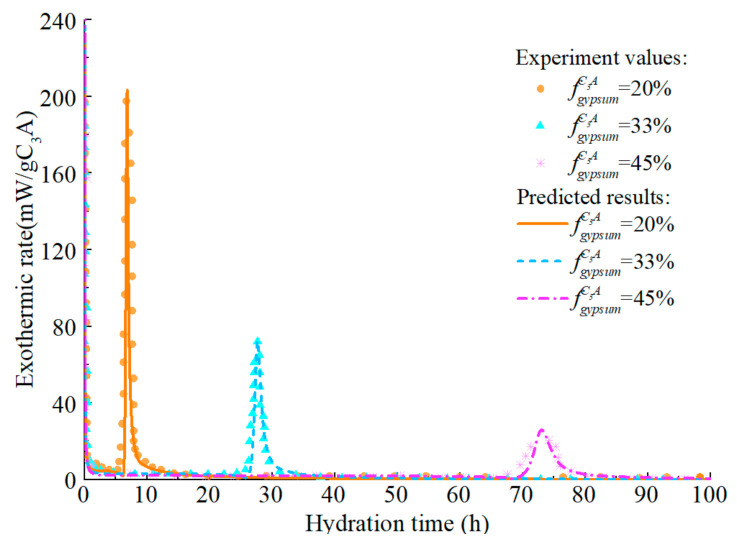
Hydration heat release rate of C_3_A in C_3_A- Gypsum system with different gypsum content (fine particle, w/c = 1.0).

**Figure 15:**
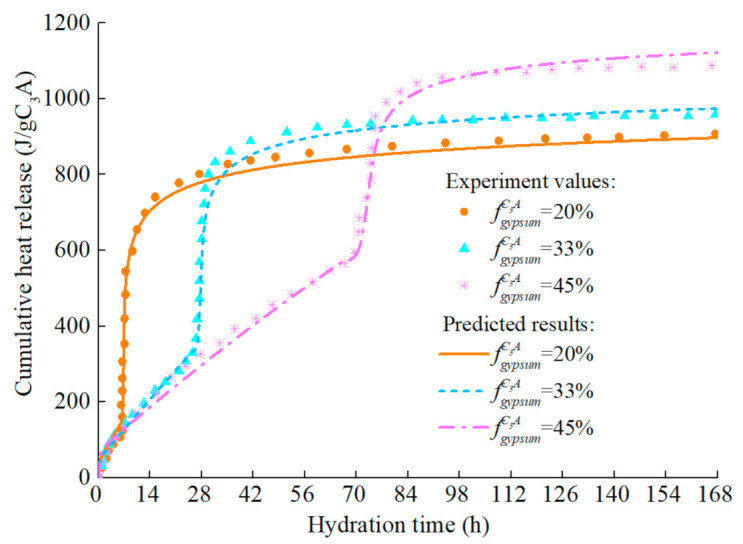
Hydration heat of C_3_A in C_3_A- gypsum system with different gypsum content (fine particle, w/c = 1.0).

The model predicts the hydration heat release rate of C_3_A (fine particles) in the C_3_A-gypsum system with different $f_{{}_{gypsum}}^{C_{3}A}$, and the experimental values and simulation results are shown in Figure 14. The increase of gypsum content in the system has different effects on each stage of C_3_A hydration. Firstly, with the increase of gypsum content, the first stage of C_3_A hydration reaction is prolonged, which is well understood. In the second stage, the main peak of hydration of C_3_A significantly widened and decreased. The reasons for this phenomenon are complex, which may be caused by the nucleation and growth mechanism of AFm [81], or it may result from the increased time needed for the removal of surface blockage of C_3_A due to the adsorption of more ions [87]. The predicted results can well reflect the above characteristics, and the predicted results are still in good agreement with the experimental values in the subsequent stages. The corresponding hydration heat curve of C_3_A-gypsum system is shown in Figure 15. The ‘step’ shaped hydration exothermic curve is significantly different from that of C_3_S and C_2_S, and the dissolution-barrier hydration model proposed in this chapter can accurately predict this exothermic process.

**Figure 16:**
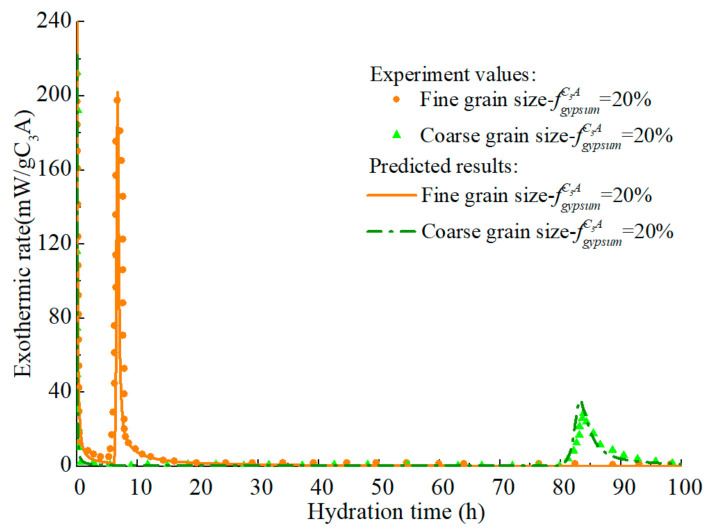
Hydration heat release rate of C3A in C3A- gypsum system with different fineness (w/c = 1.0).

**Figure 17:**
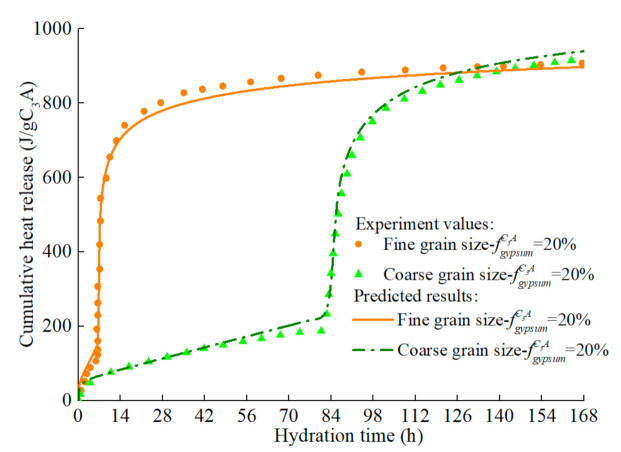
Hydration heat of C3A in C3A- gypsum system with different fineness (w/c = 1.0).

The hydration process of the C_3_A-gypsum system with the same gypsum content but different fineness is predicted and the response of the model to the particle fineness is studied. As shown in Figure 16 and Figure 17, the hydration model has a high prediction accuracy and can reflect the influence rule of particle fineness on C_3_A hydration: the coarser the C_3_A particle is, the lower the reaction rate at each stage.

**Figure 18:**
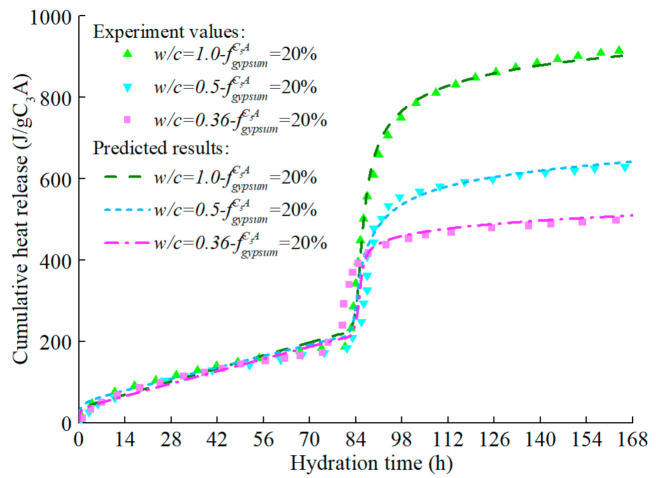
Hydration Heat of C_3_A in different Water–cement ratio C_3_A-Gypsum system (coarse particles).

Figure 18 shows the influence of water–cement ratio on the exothermic process of the C_3_A (coarse particle)-gypsum system. These results verified the previous findings [81], that is, the water–cement ratio had no obvious influence on the hydration rate of C_3_A in the first stage, nor did it affect the peak value of the main hydration peak, but it did have a certain influence on the subsequent hydration process. The total heat release of C_3_A increases with the increase of the water–cement ratio. The reasons for the above phenomena may be as follows: in the second stage of hydration, the reaction rate controlled by nucleation and growth of AFm decreases as hydration products collide. For the system with high water–cement ratio, the space between hydration products is large and the collision is not easy to occur in early age. To simulate the effect of water–cement ratio on C_3_A hydration, the coefficient $efs\left(r_{out}\right)$ representing the free hydration surface of particles is modified to approximately consider the effect of available hydration space on hydration rate

(57) $$efs^{*}\left(r_{out}\right)=efs{\left(r_{out}\right)}^{\theta_{space}}$$

(58) $$\theta_{space}=\frac{-8.93\left(w/c\right)+9.93}{1+2e^{-34{\left(\xi -\xi_{{}^{Gypsum}}^{C_{3}A}-0.24\right)}^{2}+18\left(\xi -\xi_{{}^{Gypsum}}^{C_{3}A}-0.24\right)}}$$

where $efs^{*}\left(r_{out}\right)$ represents the synergistic influence of particle free hydration surface and available hydration space on hydration rate; $\theta_{space}^{}$ is the influence coefficient of the available hydration space.

It can be concluded that after the introduction of the modified $efs^{*}\left(r_{out}\right)$, the model can accurately reflect the influence of the water–cement ratio on the hydration of C_3_A, and the simulated hydration heat release of C_3_A in each water–cement ratio system are consistent with the experimental values.

## 7. Hydration of Cement System

In the cement system, each mineral’s hydration reaction is not completely independent, and there will be a certain synergistic effect between the reactions. In the previous part, the predictive hydration models of alite, C_3_S, C_2_S, and C_3_A are put forward, and the ‘S-shape’ function is designed to quantify the hydration synergy between C_3_S and C_2_S. The activity of C_4_AF is much lower than that of C3A. For simplicity, the hydration rate of C_4_AF is set as one-fifth of the hydration rate of C_3_A in this model by referring to CEMHYD3D model [16,88]. This chapter improves the model based on the published test results to consider the synergistic hydration effect between minerals partially.

### 7.1. Hydration of Alite-Gypsum System

The hydration of alite in PC is similar to that of C_3_S, but do has certain differences. Minard et al. [89] found that the hydration reaction of C_3_S in aluminum-rich solution is relatively slow. Garrault et al. [90] measured the conductivity during the hydration process of C_3_S and alite, and found that the initial low conductivity period of alite was significantly longer than that of C_3_S, indicating the acceleration period corresponding to the growth of C-S-H was delayed. Moreover, Garralult et al. [90] confirmed that the delay of alite hydration was related to the release of aluminum in the solution by measuring the ion concentration during the first 30 min. Quennoz et al. [91] performed EDS energy spectrum analysis on alite-gypsum systerm and C_3_S-gypsum systerm, which further verified the stabilizing effect of aluminum on the alite reaction.

C_3_S and impurity alumina in alite will generate calcium aluminate hydrate, which are not good substrate for the further growth of C-S-H. It is believed that gypsum contained in PC can reacts with the alumina in alite to form ettringite, thereby removing the aluminum and increasing alite reaction rate in the acceleration period. To truly simulate the hydration process of alite in the cement system, $B_{{}_{eff}}^{}$, proposed in Section 3, is revised:

(59) $$B_{{}_{eff}}^{*}=\theta_{a}^{1}B_{0}f_{{}_{\xi }}^{*}\left(\xi \right)\frac{1}{1+{\left(\left(1-H\left(t\right)\right)/0.12\right)}^{8}}$$

(60) $$\xi_{{}_{c}}^{*}=\theta_{a}^{2}\xi_{c}$$

where the form of $f_{{}_{\xi }}^{*}\left(\xi \right)$ is consistent with the previous form, m∗ is 1.9, and the other coefficients remain unchanged; $\theta_{a}^{1}$ and $\theta_{a}^{2}$ are the influence factors of the mass fraction of gypsum $f_{{}_{gypsum}}^{alite}$ in the system on the alite hydration rate, expressed as

(61) $$\theta_{a}^{1}=4.2791{\left(f_{{}_{gypsum}}^{alite}\right)}^{0.1113}$$

(62) $$\theta_{a}^{2}=1.7432{\left(f_{{}_{gypsum}}^{alite}\right)}^{0.0406}$$

**Figure 19:**
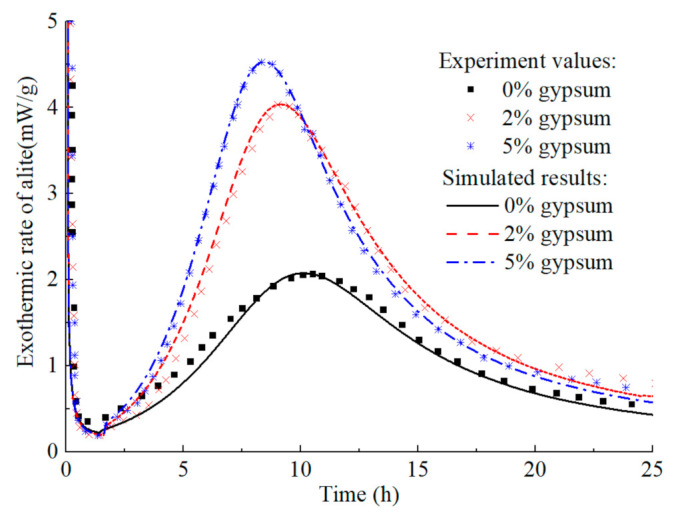
Heat release rate of alite in the alite-gypsum system with different gypsum content (w/c = 0.4).

Literature [91] measured the hydration heat release rate of the alite-gypsum system with different gypsum content. According to the particle size distribution curve, the equivalent radius of alite is approximately equal to 10.6 µm. As shown in Figure 19, after the introduction of $\theta_{a}^{1}$ and $\theta_{a}^{2}$ into the model, the revised model can reflect the influence of gypsum on the hydration reaction rate of alite.

### 7.2. Hydration of C_3_A-Alite-Gypsum System

**Figure 20:**
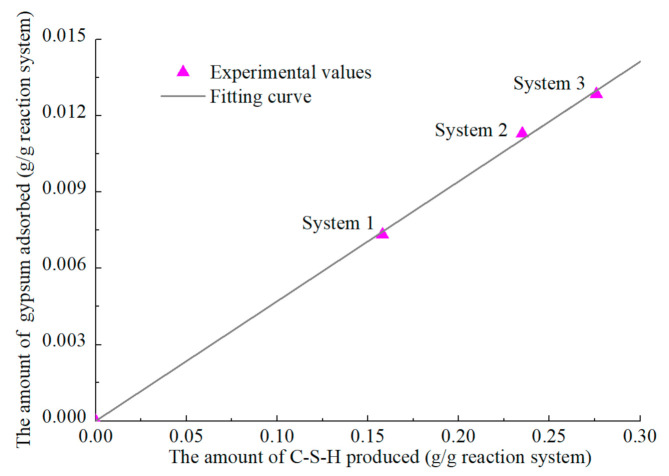
Relationship between the amount of C-S-H produced and the gypsum absorbed in the Alite-C_3_A-gypsum system.

It was found the hydration peak of C_3_A appeared earlier when alite was added to the C_3_A-gypsum system [91]. This is due to the adsorption of sulfate ions on C-S-H, resulting in less gypsum reacted with C_3_A in the first stage. In order to determine the relationship between the amount of gypsum adsorbed and C-S-H produced, it is necessary to extract the hydration degree of alite corresponding to the end of the first hydration stage of C_3_A. We calculated the amount of C-S-H produced and the amount of gypsum adsorbed in three C_3_A-alite-gypsum systems. The mass ratio of alite to C_3_A in the three systems is 92:8, and the mass ratio of C_3_A to gypsum in system 1, system 2, and system 3 is 0.7:0.3, 0.65:0.35, 0.6:0.4, respectively. As shown in Figure 20, the linear relationship between the amount of gypsum adsorbed and C-S-H produced is established by regression analysis, indicating each gram of C-S-H absorbs about 0.00471 g of gypsum, that is, $\varsigma_{{}_{G-C-S-H}}^{absorb}$ = 0.00471.

**Figure 21:**
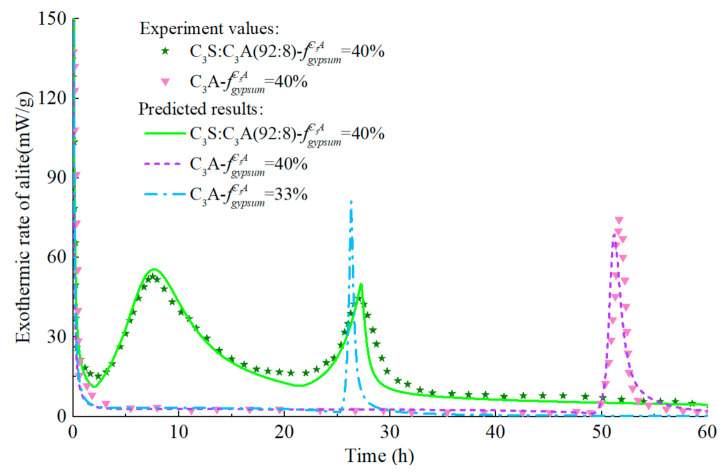
Heat release rate of C_3_A-Alite-gypsum system and alite-gypsum system (w/c = 0.4).

Firstly, the hydration heat release rate of C_3_A in the C_3_A-gypsum system with $f_{{}_{gypsum}}^{C_{3}A}$ = 40% is predicted. The w/c of the sample is 1.0, and the equivalent radius of C_3_A is about 1.8 µm. As illustrated in Figure 21, the predicted results have high accuracy, further verifying the applicability of the C_3_A hydration model. The experiment also indicated that the main calorimetric peak of C_3_A hydration widened and decreased after the addition of alite. The reason for the above phenomenon is that the matrix has been filled with C-S-H and CH before the reaction of C_3_A with ettringite. In order to characterize the filling effect of C-S-H and C-H, it is feasible to introduce a reduction coefficient into the hydration model of C_3_A

(63) $$\theta_{fill}=\frac{V^{hyd}\left(t\right)}{V_{{}_{0}}^{hyd}}$$

where $V^{hyd}\left(t\right)$ is the available hydration space volume after filling with C-S-H and CH in the system at time t, and $V_{{}_{0}}^{hyd}$ is the initial available hydration space volume.

As represented in Figure 21, the predicted curve is in good agreement with the experimental curve, indicating that the introduction of correction coefficients $\varsigma_{{}_{G-C-S-H}}^{absorb}$ and $\theta_{fill}$ enables the hydration model to consider the synergistic hydration effect of the C_3_A-alite-gypsum system to a certain extent.

### 7.3. Hydration of Portland Cement

The hydration degree and heat release rate of PC can be calculated by the equation

(64) $$\xi^{cement}=\xi^{Alite}f_{Alite}+\xi^{Belite}f_{Belite}+\xi^{C_{3}A}f_{C_{3}A}+\xi^{C_{4}AF}f_{C_{4}AF}$$

(65) $$\frac{\partial Q^{cement}}{\partial t}=\frac{\xi^{Alite}}{\partial t}Q_{com}^{Alite}f_{Alite}+\frac{\xi^{Belite}}{\partial t}Q_{com}^{Belite}f_{Belite}+\frac{\xi^{C_{3}A}}{\partial t}Q_{com}^{C_{3}A}f_{C_{3}A}+\frac{\xi^{C_{4}AF}}{\partial t}Q_{com}^{C_{4}AF}f_{C_{4}AF}$$

where *f* represents the mass fraction of each mineral in the cement; Q represents the hydration heat, while $Q_{com}^{}$ denotes the heat released by the complete hydration of a unit mass mineral.

**Figure 22:**
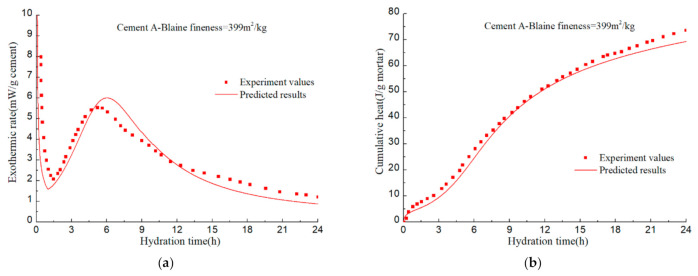
Isothermal calorimetry test and model prediction results of Cement I: (**a**) hydration heat release rate of per unit mass of cement; (**b**) cumulative hydration heat of per unit mass of mortar.

**Figure 23:**
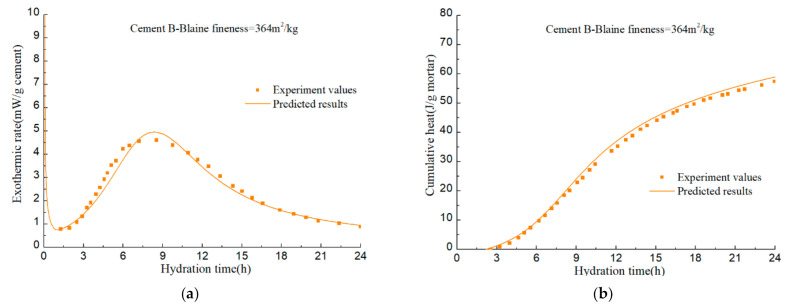
Isothermal calorimetry test values and model prediction results of Cement II: (**a**) hydration heat release rate per unit mass of cement; (**b**) cumulative hydration heat of per unit mass of mortar.

**Figure 24:**
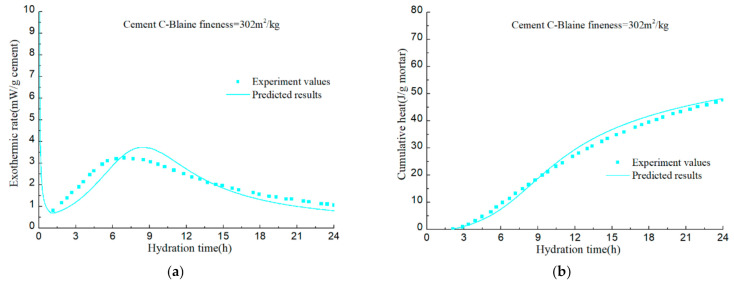
Isothermal calorimetry test values and model prediction results of Cement III: (**a**) hydration heat release rate per unit mass of cement; (**b**) cumulative hydration heat of per unit mass of mortar.

**Figure 25:**
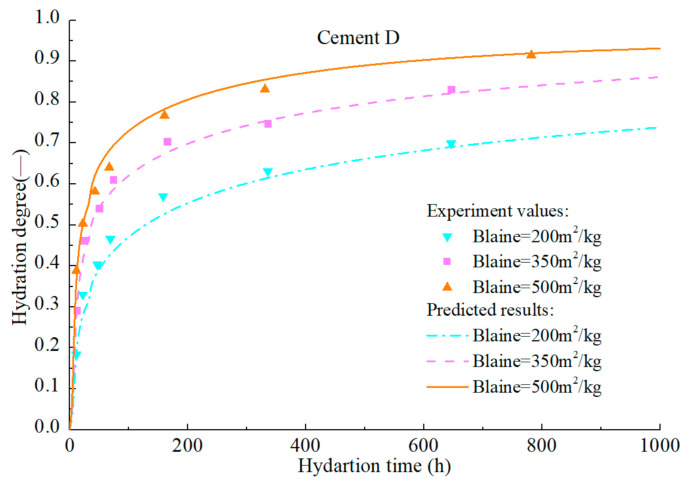
Measured and predicted hydration degree of cement D with different fineness.

**Figure 26:**
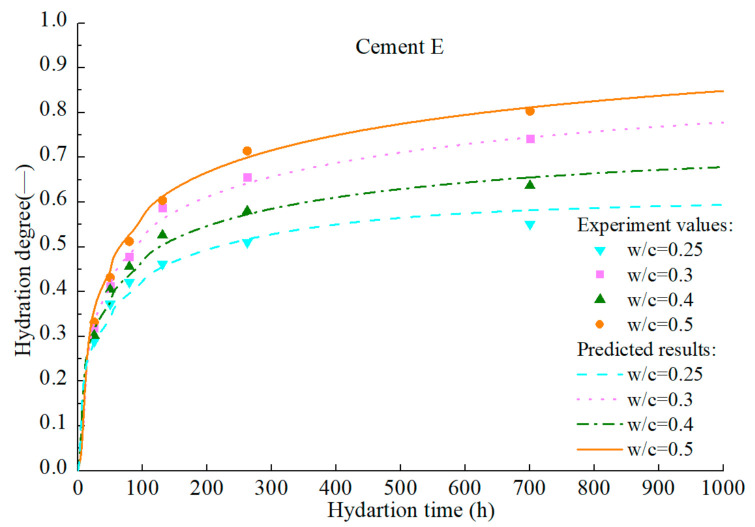
Measured and predicted hydration degree of cement H with different water–cement ratio.

To verify the accuracy of the hydration model, hydration process of various cement samples in the literatures [92,93,94] is predicted. The mineral compositions and the Blaine fineness of PCs are listed in Table 2. The water–cement ratios of cement A, B, C, and D are all 0.4. Calorimetry experiments in the literatures were conducted at room temperature of 20–23 °C. The measured values and predicted results are illustrated in Figure 22, Figure 23, Figure 24, Figure 25 and Figure 26.

The calculation results show that, given the mineral composition, particle fineness, water–cement ratio and test temperature of PC, the model can finally realize the prediction of cement hydration process on the basis of predicting the hydration process of alite, belite, C_3_A, and C_4_AF. Although certain assumptions and approximations have been made in the model, the predictive capacity of the model has demonstrated that these assumptions and approximations does not introduce significant errors.

## 8. Conclusions

In this study, the predictive hydration models of the main minerals in PC are formulated. To a certain extent the synergistic hydration effects among the minerals are considered to realize the prediction of the hydration process of PC. The major results are summarized as follows:

- Focusing on the C-S-H barrier theory, a modified hydration model of alite and C_3_S is proposed, and the accuracy of the model is verified by predicting test results. Compared with the original model, the modified model can describe the initial period and the dormant period of hydration. Moreover, by re-calibrating the model parameters, the modified model can accurately predict the long-term hydration of coarse particles.
- Considering the similarity between the hydration reaction of C_2_S and that of C_3_S, the hydration model of C_2_S is put forward, which can reflect the hydration characteristics of C_2_S, that is, the hydration rate at the early age is low, and the hydration rate at the middle and late ages is increased. By analyzing the published test results, an S-shaped function is proposed to determine the synergistic hydration effect of C_2_S and C_3_S in the system.
- The three-stage hydration model of C_3_A–gypsum systerm is developed based on the theory of dissolution and water diffusion. The three stages of hydration model correspond to the reactions of C_3_A with gypsum, ettringite, and water, respectively. The model is calibrated and validated by published test data, and can accurately predict the hydration of C_3_A in the system with different gypsum content, water–cement ratio and particle size distribution.
- Through the analysis of the published test results, a series of correction coefficients are introduced into the model to take into account the synergistic hydration effect of various minerals in the system to a certain extent. The comprehensive model shows promise in predicting the hydration process of PC.

## Figures and Tables

**Table 1:** Physical properties of various chemical components during cement hydration.

Chemical Components	Density (kg/m^3^)	Molar Mass (kg/mol)	Molar Volume (m^3^/mol)
$C_{3}S$	3150 [27,28]	0.228	7.24 × 10^−5^
$C_{2}S$	3280 [16]	0.172	5.24 × 10^−5^
$C_{3}A$	3040 [29,30]	0.270	8.88 × 10^−5^
$C_{4}\mathrm{AF}$	3770 [31]	0.486	1.29 × 10^−4^
${C\;S\;¯\;H}_{2}$	2320 [32,33]	0.172	7.41 × 10^−5^
$H_{2}O$	1000	0.018	1.80 × 10^−5^
C-S-H	2050 [18]	0.225	1.10 × 10^−4^
CH	2240 [34]	0.074	3.30 × 10^−5^
$C_{3}{\mathrm{AH}}_{6}$	2530 [35]	0.378	1.49 × 10^−4^
$C_{6}A{\bar{S}}_{3}H_{32}$	1778 [36,37]	1.254	7.05 × 10^−4^
$C_{4}A\bar{S}H_{12}$	2015 [38]	0.622	3.09 × 10^−4^
${\mathrm{FH}}_{3}$	3000 [16]	0.214	7.13 × 10^−5^

**Table 2:** Mineral compositions of PC.

Serial Number	Mineral Composition					Blaine Fineness, m^2^/kg
	C_3_S,%	C_2_S,%	C_3_A,%	C_4_AF,%	SO_3_,%	
A [92]	54.98	18.64	10.97	6.91	2.99	399
B [92]	57.51	14.73	6.60	10.68	2.86	364
C [92]	62.48	12.21	4.99	12.48	2.20	302
D [93]	71.70	5.90	9.00	10.00	2.60	250,300,500
E [94]	56.70	17.20	6.70	7.90	2.50	312

## Data Availability

Data has been presented in the paper.
